# Clinical Review, and Hospital Reports

**Published:** 1836-07-01

**Authors:** 


### Clinical Review.

### St. Thomas's Hospital.

### Clinical Reports.*

The clinical reports from this hospital, edited by Mr. South, continue to be published. The third number lies before us. The editor displays but little desire to distinguish himself in his editorial capacity. A diurnal report of a case is given, and the surgeon, or physician, or reporter appends some observations of a clinical character at its conclusion. There is little system exhibited in the pamphlet, which we fear will not prove a very lucrative speculation to those who are concerned in it. But this is their business—it is ours to present a sketch of the reports.

These are six in number. First, there is a report from Dr. Roots on Porrigo Lupinosa, and hysterical paralysis—then one from Mr. Tyrrell on compound dislocation of the clavicle backwards—then, again from Dr. Roots, on chronic dysentery—again, from Mr. Tyrrell on diseases of the joints—from Dr. Williams, on idiopathic erysipelas treated with wine—from Mr. Travers on encysted tumor, and diffused tuberculation. Such are the contents of the third number of the St. Thomas's Reports. We will select what seems to us most deserving of attention in each article.

### I. Porrigo Lupinosa.

A case of this description was admitted under the care of Dr. Roots. The patient, a man 38 years of age, had also inflammation of the chest, which was relieved by antiphlogistic treatment. Then the porrigo was taken in hand. After the crusts had been removed by poultices, the individual hairs were carefully extracted by means of tweezers, and the tar ointment was applied. Under this plan, with occasional slight alterations to meet circumstances, the patient improved so much, that for a week before his discharge from the hospital no new pustules had appeared.

Dr. Roots makes or made some clinical remarks upon the case, which are characterised by good practical sense. The gist of them may be said to be this—treat these eruptions of the scalp on rational principles. When there is much irritation, use mild or sedative applications—poultices—goulard wash or mild ointments. As soon as irritation and inflammatory action have subsided, make use of a stimulating application to the scalp. The tar ointment frequently succeeds, but, if it fails, creosote, the pyroligneous acid, solutions of nitrate of silver, and so forth, may be tried. Whatever application be resorted to, the hairs must be gently and carefully picked out of the affected parts by means of tweezers. We extract that portion of his observations which bears directly on the treatment. His description of the complaint is of course addressed to and adapted for pupils.

"As regards the plan of treatment generally, there is no one plan that you can lay down as being generally successful, but you must treat porriginous affections of the scalp precisely as you would treat any other disease for which you may be called to prescribe—namely, you must treat it according to its existing condition. Generally speaking, you will find, as a local application, the tar ointment one, I think, of the most useful, but there may be so much excitement going on in the skin, that the worst treatment you could possibly resort to would be the application of a stimulant, which the tar ointment is. If then there be much inflammation, you must make use of sedative applications, cold; perhaps the application of lead in its various forms, cold; the liquor plumbi subacetatis dilutus, or rags dipped in cold water. If the inflammation or irritation is not excessive, but still too great to admit of the

---

* St. Thomas's Hospital Reports. By John F. South, Assist. Surgeon.

use of any stimulant, then perhaps the ceratum plumbi acetatis, the ung. zinci, or a mild sedative ointment of that sort, may be the best. Supposing that there should be considerable constitutional excitement, and I have seen a few cases attended by this, then I should not hesitate to rest entirely on antiphlogistic measures; I should not hesitate where there was much fulness of habit even to take blood, under some circumstances, from the general system. But, though you might not have great constitutional excitement, you might have considerable local excitement, so much local excitement that you might not think the mere application of cold lotion or other sedatives would be sufficient to remove it. Under such circumstances you may be obliged to resort to the application of leeches; I have done so in many cases, and continued the application of leeches from time to time, in conjunction with cold lotions, until the inflammatory action has been reduced. In practice you will find that, during the treatment of this affection, you will very often have to change your plan. In the first instance, for example, you may find that a stimulating ointment may be necessary; you may employ the pure tar ointment of our Pharmacopœia, and you may find that that is not sufficiently stimulating, and you may be obliged to combine it perhaps with some other, as the ung. hydrar. nitrat., or in some instances, as I have occasionally done with advantage, the ung. sulphuris compositum of our Pharmacopœia. I have applied this for some days together, till eventually I have got more excitement in the part than I wanted. My object was to get a certain degree of excitement; this has been exceeded, and then I have been immediately obliged to change the plan of treatment, in order to subdue the degree of increased excitement which was unnecessary for the cure of the disease, and to resort, perhaps, for a few days, to sedatives merely, to ung. zinci, or plumbi acetatis, till the excitement has been partly diminished, and then perhaps again to have recourse to tar ointment, either pure or moderated by the admixture in a certain proportion with some of the ung. zinci. Generally speaking, you will find that though you may wish to produce a certain degree of excitement, yet that it will be better to do it gradually. For example, commence with an ointment somewhat of this form: one part of ung. picis, either with two or three parts of ung. zinci, and then by degrees, according to the local excitement, diminish the latter and increase the former, till perhaps you may use the ung. picis pure."

We notice this case, and these observations more particularly, because they enable us to remark, that eruptions are treated by many practitioners, in far too empirical a manner. The general principles which regulate our management of other disorders, are frequently overlooked in the instance of eruptions, and the surgeon having heard that such an application is of service for a cutaneous complaint, applies it unhesitatingly and indiscriminately, no matter what the stage or what the condition of that complaint may be. Yet the skin is perhaps more affected by varying states of system, and sympathises with other parts more closely, than any other tissue in the body. It is remarkably prone to alterations of vascularity, and to inflammatory action; and when inflamed, it is just as likely to be injured by irritating applications, as the lungs, or the liver, or any membrane, if exposed, would be.

The important rule in the treatment of eruptions, is, to subdue inflammatory action, before specific remedies are used. Whether the disease be porrigo, or eczema, an exanthema or a trivial patch of herpes, this principle of treatment must be acted on. Of course the amount of depletory measures, and the nature of the sedative, must be determined by the state of the particular case, and the knowledge and judgment of the surgeon.

### II. Hysterical Paralysis.

The case we will now introduce is an important one—important, at least, to him, who is of opinion that the real use of physic is to understand disease and cure it.

*Case.* A married woman, æt. 24, admitted Sept. 12th, 1835, under the care of Dr. Roots.

States that four years ago she had an attack similar to the present, but regained perfect power over the side affected. About twelve days since she was seized with great pain in the head, and vomited about a table-spoonful of coagulated blood. The pain in the head having continued two days, whilst sitting on her bed, she suddenly fell down insensible. She continued in this state for twelve hours, at the end of which period she recovered her senses, but the right side of her body was paralysed. For a long time she was unable to speak, and the pain in the head remained unabated.

On admission, the right arm and leg were perfectly paralysed, but the face was not implicated in the attack. The pain in the head continued, though somewhat less intense in character. The sounds of the heart were natural. Pulse 75, small. She was low-spirited, cried at times, and complained of a frequent sensation of choking; having, also, tenderness of the epigastrium.—Power over the bladder remained perfect.

The treatment consisted in the use of the hyd. c̄ cretâ—leeches to the epigastrium—and milk diet. The pain in the head diminished, and by the 18th, she had regained some power over the paralysed arm. But she now suffered from globus hystericus, and palpitations. The pulse was 78.

On the 22d, the pulse was 86. On the 26th, it was 98. She could move the arm much better, but had no power over the leg. She fainted almost every day. *Diæta sicca.*

29th. *Hirud.* xvij. *epigast.* *Quart of beef-tea daily.*

On the 2d of October, she had perfect power over the arm—there was still some pain in the head and drowsiness. Pulse 100.

*Omit. Hyd.* c̄ *Cret. Emp. canth. inter scapulas.* ℞. *Infus. valer.* ℥iss. *Sod. carb.* ℈j. *Sp. ammon. arom.* ʒj. *Tinct. hyos.* ℥ss. *ter die.*

On the 6th she felt better, but had no power over the leg, which, she said, felt numbed.

*C. c. ad* ℥x. *regioni sacral.*

On the 9th she was put on ordinary diet. On the 13th, she was able to move the toes of the affected leg. She was ordered a pint of porter daily. On the 16th, she could walk without assistance. Frictions with mustard were prescribed, and a mixture containing quinine was given. On the 27th, she could walk without assistance. The uterus continued inactive. On the 6th of November, after a fright, she had several fainting fits, and complained of much pain in the lower part of the back. For this she was cupped over the sacrum. On the 17th, she could walk nearly as well as ever. She had still occasional fits of hysteria.

Dr. Roots makes a great many observations on this interesting case. We will notice those which bear immediately on its more important features.

"You will remember," says the Doctor to his class, "I related to you, that, four years before she said she had been the subject of a similar attack to the one for which she was admitted. How long it lasted I do not exactly know, because it is not noted down; but it is quite certain that she recovered perfectly the use of the affected side.

With regard to the symptoms, when she was attacked twelve days before her admission, she complained of great pain in the head, and stated she had vomited a table-spoonful of blood. Whether she did or did not, I do not know; there was no proof of it; but, as it is asserted, I am bound to believe that she did vomit some blood. The pain of the head continued for two days, and then she fell into what is termed *a fit,* and remained insensible for twelve hours. During these twelve hours it is stated she remained perfectly still. On arousing from this state, she could not speak for some time after. However, it does not appear that much, or even any thing was done, but the power of speech returned, leaving behind not the slightest vestige of paralysis about the muscles of the face, or of the tongue. But at the same time that she lost the power of speech, she lost the power of volition over the right arm and the right leg, and also the power of sensation in them. In conjunction with these, you are to remember that she was low-spirited; that she cried at times; that she occasionally fainted; and complained of a choking pain in the throat, and of pain at the epigastrium.

What was the condition of the brain, or what was the condition of a portion of the nervous system, which gave rise to this paralysis? It is quite clear that it could not be connected with any thing like effusion; it is equally clear to my mind that it was not connected with any thing like inflammation. It is possible, nay, it is probable, that it was connected with some degree of congestion in a portion of the blood-vessels, which might produce some pressure; some impediment to the function of the nerves, both as regards volition and sensation. It is probable, that during the time that she was lying in a state of insensibility, having lost the power of speech, there was congestion in the vessels of the brain; and that congestion—supposing the state to have arisen from congestion,—must have speedily given way of itself, because there is no proof of any thing being done to relieve her, and, consequently, there could have been no effusion; for you cannot imagine that effusion could have been absorbed so rapidly: neither can you imagine that there was inflammation, because inflammation is not likely to have given way without some active treatment. One, perhaps, in my mind, of the strongest reasons for imagining that there was congestion to a certain extent is this, that, tracing the history of the case before I saw her, I find she was leeched altogether three times during a period of between a fortnight and three weeks, and the pulse, when she came in, is stated to have been 75, but feeble: it remained 75, after even the first application of leeches to the epigastrium; after the application of the second, it became 78; from 78 it soon got up to 86, to 98, and to 100; and as the pulse became quicker, so did the paralysis of the arm diminish."

We think there can be no question of the rationality of Dr. Roots' opinion. That the case was one of those, in which that state of system that we call hysterical was at the bottom of the symptoms, is rendered more than probable by all the circumstances. The age of the patient—a former similar attack leaving no injurious effects behind it—the presence of amenorrhœa, and of decided hysterical symptoms, as globus—the negative proof derived from the absence of distinct symptoms of inflammation of the brain or of effusion on it—and, finally, the effects of treatment, are all characters that mark with tolerable clearness the nature of the malady.

But in hysterical affections it is not unfrequent for local congestions to occur. The constitution of the female, in whom the circulation is periodically disturbed, renders her particularly prone to these irregular determinations of blood, which, aided by an excitable nervous system, produce such strange and undefined disorders.

The cautious surgeon or physician, aware that these congestions sometimes tremble on the brink of inflammation, will, under circumstances calculated to awake suspicion, or in plethoric habits, take care to act on the safe side, and to employ topical depletion. But he knows that depletion must not be carried far, and in most cases must not be resorted to at all, lest it aggravate that state of nervous irritability, which is frequently the cause and always the concomitant of the other symptoms.

There are one or two points in the present case which should not be dismissed unnoticed.

"It was curious," says Dr. Roots, "to observe, with regard to this woman, a fact which is not noticed in the narration of the case, but which I will mention—how strong the influence of the mind was in aiding her recovery. For a length of time she was not able to do more than to move her toes, and gradually to draw up her leg. She seemed afraid; she had persuaded herself that she was perfectly incapable of doing it; but, on insisting that she should quit her bed and attempt to stand, telling her that she should be supported by people in the ward, she found that she really was capable of doing more than she had imagined. Still, there was that degree of hesitation, that degree of trepidation, in attempting to use the limb, which made it plain that it was the result, to a considerable extent, of mere fear. In order, then, to work upon her mind, I requested Mr. Clark, who has had the care of her as my clinical clerk, in her hearing, that if in two days no manifest improvement had taken place, he would put on a good blister over the loins, and over the right hip. I desired him not to put it on unnecessarily, but if he found no gradual improvement, then to resort to the blister. By means of the strong impression thus produced, at least, I am firmly persuaded such was the reason she day by day improved, without the necessity of applying it all."

It is a fact well known to the practical man, that whether there be or be not deceit on the part of hysterical patients, their symptoms are much under the control of powerful mental impressions. How constantly does the threat of a bucket of cold water arrest a hysterical fit. The dread of a caustic issue will sometimes cure hysterical pain in the knee. At the Hôtel Dieu M. Dupuytren was in the habit of observing in the hearing of his hysterical patients, that, unless there was improvement by a certain day, he should be under the necessity of applying the actual cautery. That was always an effectual hint.

It is not only charitable, but, from what we know, it is most reasonable to suppose, that in the majority of cases, the patient is under the influence of a feeling that her ordinary efforts are unable to control, and that only an unusually violent influence is adequate to give her the mastery over it. We see so many instances of educated, religious, and highly moral females, who are evidently anxious to escape from suffering, displaying at the same time such hysterical extravagances, as might appear the offspring of positive deception. But all the circumstances, are, in most cases, so opposed to the truth of this idea, that he must be an ignorant as well as a malicious man who can indulge it.

But it must be owned that in some instances there are actual falsehood and trickery at the bottom of the patient's complaints. This unworthy conduct is not confined to the lower class of females, but is occasionally seen in those whose station and whose education too, should induce them to disdain to practice such mean artifices. We have known many instances of retention of urine feigned by females, in order that the catheter might be introduced for them. There have been instances also, of young ladies putting common garden sand in their chamber-pots, in order that they might appear to have been voiding gravel. Sir Benjamin Brodie is in the habit of mentioning a case that occurred to him some years ago, in which a palpable trick was detected. He was attending a young lady of distinction, for pain in the knee of a hysterical character, but attended with such swelling as induced him to suppose that there must be some structural mischief. The means he adopted seemed of little service. One night he was summoned to the young lady in haste, for some nervous symptoms, and he accidentally looked at the leg. He was a little astonished to find a ligature tied round the thigh, so tightly as to occasion the swelling below it, that had puzzled him. It turned out that the lady tied this kind of garter round her thigh every night, in order that her leg might be subjected to examination in the morning.

When we look at the manner in which hysteria sometimes slides into mania, and reflect on its connexion with satyriasis, and chorea, and chlorosis, we may admit that even in such gross cases of malingering as this, the individual is impelled by an almost uncontrollable impulse. The ordinary motives of action are inadequate to explain her disgraceful conduct, and the philosopher must allow, that although, for the sake of morality and decency, such behaviour should be reprobated and put down by chastisement, yet Nature pleads in *his* ear her excuse. The interests of society, and of the female herself, require that that excuse should not be listened to. In this, as in some of the forms of monomania, more good results from considering the individual as strictly responsible for actions, than from yielding to the plausible apology of reason. It is certain that strong sentiments, whether of hope or fear, of duties to be performed or of disgrace to be encountered, will always enable the individual to curb, and not unfrequently to subdue, the tendency to impropriety.

2. The mode in which the paralysis declined is properly adverted to by Dr. Roots.

" I am quite sure every one is aware, that in paralysis arising from any effusion having taken place in the brain, either from simple effusion or from rupture of a vessel in the brain, that the arm, the nearest point to the seat of disease, is the last that recovers. You rarely see hemiplegia arising from effusion taking place in a portion of the brain, I can almost say *never;* at least I do not myself ever remember to have seen it: but the recovery of the lower extremity long precedes that of the upper, and very often nearly perfect recovery of the lower extremity takes place, while there is not, perhaps, even as long as the patient lives, the slightest improvement in the upper extremity. I was quite clear then, even from this circumstance, that there was no mischief going on or existing in the brain."

Dr. Roots makes some remarks on the treatment of hysteria, which do not seem to require particular notice. We therefore pass to the succeeding case.

### III. COMPOUND DISLOCATION OF THE CLAVICLE, BACKWARDS.

This case may be quickly dispatched. The patient was an excavator, and the accident happened from earth falling in upon him, and driving a pick-axe against his chest. The sternal end of the clavicle was dislocated backwards, so that it lay on the trachea, behind the first bone of the sternum. The wound was extensive. A back-board with straps to keep the shoulder back, was sufficient to re-adjust the bone, and the patient did extremely well.

### IV. CHRONIC DYSENTERY.

The diurnal details of a case of this kind are related. But they are tedious, and the following abstract of the chief particulars, presented by Dr. Roots in his Clinical Lecture, presents a correct and sufficient account of them.

The patient was a sailor, aged 39, just returned from the South Seas. He had been exposed on his voyage home to hard weather and bad food, and his complaint had commenced three months prior to his entrance into the hospital. He then had the usual symptoms of dysentery.

" The first part," says Dr. Roots, " of this man's treatment—during the first twenty-six days—consisted in the application of leeches; in the exhibition of starch and opium injections, with the occasional exhibition of a dose of castor oil, and a very limited mucilaginous, farinaceous diet. My reason for ordering the leeches must be apparent. It was quite clear he was suffering under chronic inflammation of the mucous membrane of the large intestines, and I considered that chronic inflammation possessed too much activity of character to permit my depending upon opium, or astringents, or mercury alone. My reason for ordering him to take hydrargyr. c̄ creta was, that he had been in a hot climate, that there seemed to be evidently functional derangement, at least about his liver, and I ordered him the mercury rather for the purpose of obtaining a more healthy condition of the liver, than with the idea of curing the chronic inflammation of the bowels. The starch and opium injections were, of course, merely directed to be given for the purpose of relieving the tenesmus, and to some extent the tormina. The castor oil was exhibited, because one of the common symptoms in dysenteric affections, whether acute or chronic, but always in the acute form, and occasionally to some extent in the chronic form of the disease, is, that a portion of the feculent matter is retained. There is spasmodic action of the muscular fibres of the bowel, and though there is great griping, and frequent calls to relieve the bowels, yet the feculent matter is often retained, and becomes an additional source of irritation; and, therefore, likely to increase the inflammation.

You will observe that, under this plan of treatment, with a grain of opium every six hours, the symptoms were to a certain extent alleviated; he had better secretions, he had less tormina, less tenesmus. But the mercury did not cure him; though he was kept under its influence for nearly twenty-eight days, yet still the irritation of the mucous membrane of the bowel was not at all diminished. It was then, that having lessened by means of leeches and blisters the inflammatory action of the bowel, that having produced better secretions from it, and a more healthy secretion of the liver, I thought I might begin to enter upon the exhibition of a more powerful astringent than opium, and therefore it was I combined the vegetable astringent of kino with opium and the sulphate of copper. You saw from the case he was again somewhat relieved, though still the bowels were too irritable, and it was speedily necessary to increase the dose of compound kino powder to twelve grains; and at the same time I ventured to increase the dose of sulphate of copper to three-fourths of a grain. This the stomach would not bear—vomiting was produced—and therefore it was speedily diminished to half a grain; and at the same time the dose of compound kino powder was increased to a scruple. Sulphate of iron was afterwards ordered in conjunction. My reason for ordering the latter was, that, from his generally debilitated condition, I thought he would bear a tonic of that kind, experience having told me that similar cases had often been advantaged by it. From that time, for a period of ten days, he certainly seemed better when taking the iron; but again the symptoms recurred, again irritation was produced, he could not bear even half a grain of sulphate of copper; nay, it was with difficulty that a quarter of a grain was retained on the stomach, except through the aid of hydrocyanic acid. But though the hydrocyanic acid did enable him to bear that small quantity of the sulphate of copper, yet still the irritability of the bowels was not diminished.

These mineral astringents, sulphate of copper and sulphate of iron, (for the sulphate of iron is possessed to a certain extent of some degree of astringency,) in combination with the opium, the irritability likely to be produced on the stomach being also prevented for a time by the exhibition of hydrocyanic acid, still were unable to relieve the irritability of the mucous membrane of the bowels, and therefore it was I determined upon trying the nux vomica. I did not give the nux vomica in what I consider a very large dose, because I have given as much as fifteen, eighteen, or twenty grains every six hours. I have given it to out-patients of this hospital; I have given it repeatedly in St. Pancras Infirmary, without finding it produce those constitutional effects which it did in this man. However, in consequence of the twitching and spasmodic action of the muscles, I abandoned it at the end of two or three days. Then it was that, finding the mineral astringents had not produced any real benefit upon the man, I had recourse to a very slightly-increased dose of compound kino powder, that is, from twenty to twenty-five grains every six hours, and giving him at the same time another very powerful vegetable astringent—the infusion of galls. Under this simple plan of vegetable astringents the man got entirely well."

Dr. Roots makes several judicious observations on the character, and pathology of dysentery. Being addressed to students, they are, of course, of an elementary nature, and require no notice from us. The only point to which we think it necessary to advert, is to our author's experience on the best mode of managing this troublesome disorder.

When there are pyrexia and much pain, he employs, as all good physicians do, local depletion by means of leeches and counter-irritation. He has found blisters most useful; but the tartar-emetic ointment has also been of service.

The warm bath is beneficial.

Dr. Roots has never seen mercury cure dysentery. He appears to give it merely with the view of producing a better secretion and better condition of the liver. Few practical physicians believe that mercury will remove chronic dysentery in such a climate as this. Its employment in India is another thing, for all the circumstances there are different. Yet at the same time we think that Dr. Roots has rated it too low in this country. When we reflect on the influence that mercury exerts on the mucous membrane of the alimentary canal—on its power of restoring or of altering its secretions, as well as the secretion of the liver—on the fact that in dysentery the functions of the mucous membrane are greatly disturbed—on the frequent complication of much inflammatory action with the slighter dysenteric affection—when we reflect on these circumstances it seems reasonable to suppose that mercury would be, at least occasionally, useful. We think that the experience of most medical men is in its favour—not as absolutely curing dysentery, but as adapted for its earlier and more inflammatory stage—preparing the way for astringents, and rendering their employment more safe.

Opium, in one form or other, and to a greater or a less extent, is indispensable.

Astringents are extremely useful. Of course they are adapted for the later stage of the complaint, after the inflammation and the severer spasm are relieved. Astringents may be exhibited even when it is necessary to leech or blister, in consequence of pain and tenderness. But if much excitement is re-established, the astringents must of course be discontinued, till that has been removed. Dr. Roots speaks judiciously of the sulphate of copper. He does not affect to have found it always successful; on the contrary, he admits that he has often seen it fail. This agrees with our own experience. Neither does he pretend that it is a tonic—quite the contrary. In this case the compound kino powder, in the dose of twenty-five grains every six hours, and conjoined with galls were successful; but Dr. Roots properly observes that, although a particular astringent may succeed in one case, it does not follow that it will answer in another. The following are his remarks on the infusion of galls.

"With respect to the power of the infusion of galls, I remember the first case in which I ever saw it exhibited was whilst I was Assistant Physician to this hospital. It was a case which my friend Dr. Elliotson requested me to see, indeed, it occurred in his own coachman. Astringents to a great extent, and if I remember right, sulphate of copper, had been given without effect. We agreed between us that we would give the infusion of galls with opium, and it was taken with the most marked beneficial result: since that time I have continued occasionally to use it when other remedies have failed, and with considerable advantage. It is a very nauseous medicine, and there are few patients who will submit to take it. I had a patient in private practice, a lady, who suffered under chronic inflammation of the mucous tissue of the bowel, of long standing, who attempted to take this remedy, but after the third dose, she said that, in point of fact, she would rather die than take such nauseous medicine."

### V. Diseases of the Joints.

A series of cases of diseases of the joints are reported. We have at different times, of late, adverted so fully to this class of maladies that it would be unprofitable to allude to them again at present. The majority of the cases before us present indeed no particular features of interest, with the exception of one, to which we shall direct attention.

Case. *Supposed Inflammation of the Internal Lateral Ligament of the Knee.*

Harriet Wynch, æt. 39, a cook, admitted Sept. 4th, 1835, under the care of Mr. Tyrrell.

States that about two months ago, after much exercise, she experienced pain at the posterior part of the left knee, which shortly became so severe, as to compel her to leave work. She then went into the country, and by rest and quiet, the pain having subsided, she returned to her situation; but in the course of a few days, the pain recurred with increased violence, and was particularly severe during the night, so as to materially disturb her rest.—Leeches were applied, but without benefit.

When admitted, she complained of a dull aching pain at the posterior and inner part of the knee, especially towards evening, and during the night. The posterior part of the joint was somewhat swollen, and extremely tender.

*Hirud.* xxiv. *genu. Hyd. sub.* gr. j. *omni nocte.*

The leeches relieved the pain in the knee for a short time, and she was subsequently cupped over the vastus internus muscle, but with little benefit. The pain at night continued severe, and disturbed her rest. Opium was exhibited, first in the dose of half a grain, then in that of a grain every night. It gave her more rest, but occasioned headache and costiveness. In consequence of the continuance of headache, the opium was given up, and the galbanum pill with henbane substituted for it. We should observe that two blisters, and mercurial ointment had been applied to the knee. The subsequent reports we will extract.

" Nov. 4.—She rests better at night, but complains of more pain in the knee, the inner part of which is very tender. *Capt. Pil. bis quotid. Applic. Cerat. Sapon. et Opii part. æqual. genu.*

Nov. 11.—Sleep undisturbed, and says she has less pain in her knee than since her admission; but pressure on the inside of the joint still gives pain.

Nov. 18.—The pain in the knee very severe last night, and the joint very tender. ℞. *Tinct. Valer. Ammon.* ʒj. *Aq.* ʒxj. *bis die.*

Nov. 25.—Much the same, but complains that her rest is again disturbed by pain in the knee. She has frequent tremors of the limb, lasting about ten minutes, after which the pain is very much increased. The whole joint is tender, especially at its inner and posterior part, to which the pain is principally referred. Headache at times very distressing. ℞. *Ferr. subcarb.* ʒss. *ter die.*

Dec. 2.—Pain and tenderness of the knee much the same. Complains of throbbing in the joint, and has occasionally extreme heat on its inner side, continuing for about a quarter of an hour, and followed by a corresponding degree of coldness. Trembling of the limb occurs more frequently, and the pain is greatly increased for a short time after its cessation. The headache at times very distressing, and chiefly referred to the forehead and temples. Appetite impaired. Alvine secretions of a very dark colour, and exceedingly offensive, with torpor of the bowels. Uterine functions natural. ℞. *Assafœt. Gum.* ʒss. *Aq. Fervent.* ℥viij. *ft. Enema tepid. alt. noct. injiciend.*

Dec. 8.—Complains of tightness across the chest and cough; rest at night still much disturbed;—headache relieved;—secretions natural, and appetite improved. The knee remains in the same state.

Dec. 10.—Left the hospital to-day, and directed to take the following, instead of the former medicine. ℞. *Ext. Coloc. c.* ℈j. *Ext. Hyoscyam.* ℈ss. *Ext. Aloes c.* gr. jss. *divide in pilulas* vj. *quar. capt. duo alt. noct. Mist. ferr. c.* Oj. *Tinct. Castor.* ℥j. *fiat Mist. cujus capt. cochl. duo ampl. bis die.*"

The following are the observations of Mr. Tyrrell upon the case.

" The case of Harriet Wynch (page 307) has been an interesting one, inasmuch as, in the first instance, there were symptoms indicating acute inflammation of the internal lateral and posterior ligaments. Perhaps the situation in which the affection was described by her to exist, led me not to go so far into the history of the case as I might otherwise have done, and this, in combination with the description that I

misunderstood, that she had been relieved by counter-irritants and leeches, led me to presume that it was a case of common inflammation of these ligaments, which would be relieved again, I considered, by rest and the treatment from which she had previously derived benefit. I, therefore, in the first instance, attended to the secretions, and in consequence of there being a great deal of tenderness, employed local bleeding, first leeches, and afterwards cupping, and then had recourse to counter-irritation by a blister. The little amendment however that took place, and more particularly the description of the pain, induced me to look a little farther, and I then found that she had the nervous, tremulous pulse belonging to hysterical patients; that she was frequently the subject of headache, seldom without it; and that this was principally complained of over the forehead. This, however, was a little puzzling, when I came to find that the uterine secretions were in a proper condition: but as I have frequently seen hysteria in a violent degree while the uterine functions appeared good, I was not so much surprised by observing this. I turned my attention more particularly to the general treatment: locally, I applied some ointment and fomentations: but in spite of the remedies adopted, very little progress was made. The fact is, she was apparently losing flesh, and had become more pallid than when she was admitted; she had a weaker pulse, and altogether less power and more headache, although, at times, the headache was slightly improved; she had that peculiar tremor which often attacks persons disposed to hysteria; and other symptoms affecting the knee, which was not so well." "She complained, perhaps, more acutely, if you pressed on the lateral ligament, or the posterior part of the joint; but that is not a matter of surprise.

Supposing that there had been actual ligamentous disease, what would have been likely to occur under the improved condition of the general health; that is, losing power and becoming more irritable? I know if there had been any fibrous disease, what would have been the consequence. Instead of a mere thickening from the deposition of adhesive matter, we should have had ulceration take place, evinced by a further tumefaction, by an increase of pain, by general rigors indicating the commencement of suppuration, and then we should have had the disease go on in this way, but instead of which, you find that one day the report is, the knee is rather better, the pain less, and she rests better; and then a day or two afterwards, without any obvious reason, the pain is aggravated, and the local disease increased: thus there are frequent changes. This is not the character of actual disease of the ligaments; it does not change thus, unless there be some alteration of constitution from time to time, which will enable you to account for the local changes which take place."

If reported cases are to be of service to the public, two things are essential on the part of the reporter—accurate and discriminating observation, and fidelity of description. It is obvious that many facts derive their whole utility and value from the discrimination in them of nice particulars. Two men, for example, may have sores upon the penis. In one, the sore may have a granulating surface, and a thickened substratum and edge. Mercury cures it. In the other individual, the sore has a dark and sloughy surface, no thickening beneath nor around it, and an excavated character. Mercury makes it spread, and it heals under the exhibition of bark and stimulants. If a narrator of these two cases contented himself with saying that each patient had a sore upon the penis, and that in one it was small and not painful—in the other large and very tender, and then were to observe that mercury proved useful in the first, and injurious in the second, it is clear that the reader would obtain no precise information of the causes which rendered its effects in the two cases different.

The cases in the Reports before us have been drawn up by pupils, some of whom appear to be indifferently qualified for executing the task with success. In the present instance, the reporter is obviously unacquainted with the characteristics of the case he attempts to report, and his report is, consequently, next to useless.

The matter stands thus. Sir Benjamin Brodie (for we are not aware of any surgeon having accurately described the case before him) has pointed out a peculiar affection, which occurs chiefly in hysterical or nervous females, and which is liable to be mistaken for serious disease of the articulation. The patient complains of excessive pain in a joint. There is usually but little, if any swelling. The pain is aggravated by motion, and by examination of any kind. These symptoms are common to several diseases of the joint. They may occur from ulceration of the cartilage, or from inflammation of the bone. There is, therefore nothing distinctive in such symptoms; and were a surgeon to publish a case which presented them, and say that they did not depend on ulcerated cartilage or inflamed bone, we must take his word for it. But Sir Benjamin Brodie did not leave the matter thus uncertain. He remarked that the pain, in these cases of nervous affection, differs from that in organic disease in the following respects.

First, it is unnaturally violent, being produced by causes too slight to affect inflamed cartilage or bone. Thus, the slightest tap upon the skin, or pinching it, will produce excessive pain; more, perhaps, than even pressing the ends of the bones together.

Secondly, the pain may exist remotely from the diseased parts, and in spots which have no sympathy with them. The pain is often, in short, very universal. If the knee is affected, the patient will frequently cry out with pain if you touch the skin of the foot. And she will be pretty sure to do this if her attention is directed to it, and if the surgeon observes in her hearing, that that is always a symptom of her complaint.

Thirdly, if the patient's attention is drawn off, the surgeon may do, unnoticed, all that occasioned such apparently intense suffering but the minute before.

Fourthly, there are the general evidences of hysteria, or of a nervous habit, and commonly the disturbance of the general health is neither of the kind nor the degree which would accompany serious organic disease.

Now, a case drawn up with the view of shewing that nervous symptoms may ape inflammation of the ligaments of the knee-joint, should contain, at least, an allusion to the presence or the absence of such distinctive characters as the preceding. Yet no such allusion, or scarcely any, is contained in the report, which is thus defective in the most essential particulars. Mr. Tyrrell, indeed, in his clinical remarks, very properly supplies in some measure this defect, and observes :—

"The point which convinced me that the affection was not one of actual inflammation of the ligaments of the knee, but a sympathetic disease depending on the peculiar condition of the constitution is this, that if she had inflammation merely of the internal lateral ligament and the posterior ligament, the pain would be confined to these parts, particularly when the limb was at rest, and she would complain of pain, particularly on pressure on those parts; but when you examined the surface of the joint, it mattered little where you pressed, if on the patella, on the ligamentum patellæ, on the space between the internal lateral ligament and the ligamentum patellæ, on the vastus internus, or down below this muscle on the head of the tibia, it was all the same: she complained of pain."

This is a point which is not even hinted at in the report—an inexcusable omission. We are sorry to observe that many of the cases reported by students are by no means calculated to enhance their reputation. They are done in a slip-slop and tiresome way, which, however it may suit a ward-book, is not well calculated for publication.

### VI. Idiopathic Erysipelas, treated with Wine.

Dr. Williams relates three cases, and presents fifteen pages of remarks, for the purpose of shewing that idiopathic erysipelas may be treated with wine.

As the stimulating practice is old, and now nearly exploded, and the cases in which it has been adopted numerous, we shall only extract one from the small collection before us. That one is—

*Case.* "Peter Westneat, æt. 18, admitted into King's Ward, Jan. 21st.—States, that four days ago he was exposed to intense cold for some considerable time. In the evening of the same day he was attacked with rigors, sickness and headache, and on the following day with erysipelas of the face. When admitted the whole face was tinged; the eyes completely closed, and the cheeks vesicated. He complains of a sense of tingling and heat in the parts, with distressing headache; his sleep is disturbed; his respiration hurried; pulse 140, full and soft; great heat of skin; tongue foul and dry; bowels rather confined. ℞. *Pulv. Rhei,* c̄ *Hydr.* ℈j. *stat. Mist. Senn. c. postea.*

Jan. 22. *Vin. rubr.* ℥jv. *indies. Sago—Maranta.*

Jan. 23.—No increase of the local mischief: tongue less dry; pulse 120.

Jan 25.—Swelling of the face subsiding; tongue moist, but loaded; bowels act daily; pulse 108.

Jan. 27.—Expresses himself better in every respect.

Jan. 30.—Improving: the face but little swollen, and the cuticle desquamating; pulse 90.

Feb. 12.—Discharged quite well."

We will, for the present, leave the case, and dodge Dr. Williams through his observations, which are rather devious. He seems to have none of that spirit of doubt, which, by undecided persons, is considered philosophical, but expresses his opinions with the positiveness which becomes the professors of an exact science like our's. Dr. Johnson liked "a good hater;" and Dr. Williams seems to like a good believer—for thus he starts with erecting his creed.

"One party contends that it (erysipelas) is a disease of simple inflammation, and ought to be treated like the other phlegmasiæ, namely, by general or local bleeding. On the contrary, another party, having no theory to support, affirm that a long experience has shewn that bleeding is often highly injurious in the treatment of erysipelas, and that an opposite or tonic mode of treatment is much more uniformly successful. I entertain the latter opinion, and I think it can be supported both by fact and by argument."

The circumstance of Dr. Williams so decidedly supporting the latter party, must materially increase their chances of ultimate success. He adopts the following line of argument. Inflammation, he says, may be produced by mechanical or chemical causes, or by morbid poisons. In the first class are the phlegmasiæ, which require for their treatment active antiphlogistic measures—in the second, various fevers, which a long experience has shewn to be aggravated by bleeding.

But Dr. Williams affirms that erysipelas is also the product of a morbid poison, and a contagious and infectious disease; and, consequently, that bleeding cannot be the rule of its treatment. When he says it is contagious, he is in earnest; for, if we took his opinions au pied de la lettre, erysipelas would be a vast deal worse than the plague which has broken out amongst the men-milliners in Tottenham Court Road. For example, Dr. Williams informs us that, so satisfied of the contagiousness of erysipelas are the medical officers of St. Thomas's Hospital, that they recommended the destruction of some adjoining houses; and a month scarcely elapses without some new and striking instance of it. A case, he says, has just occurred in King's Ward, where a patient of Dr. Roots', who had been some weeks in the house, was seized with erysipelas of the head and face very shortly after the introduction of two cases labouring under that disease into the ward. A fact which, as erysipelas is generally disposed to prevail at certain periods, may possibly not be deemed conclusive.

Dr. Williams, from his experience in St. Thomas's, has even been enabled "to assign some of the laws of the erysipelatous miasmata." It is capable of diffusion through the atmosphere, and Dr. Williams informs us that it may go fifteen feet.

The contagion of erysipelas also infects substances that have been in contact with the patient's person, or fomites.

And, finally, the poison of erysipelas "probably lies latent from a few hours to about ten days," though the period is not accurately determined.

Such are the laws of the contagion of erysipelas assigned by Dr. Williams. It must be owned, that, considering how many persons doubt if erysipelas is contagious at all, they are not deficient in precision.

Dr. Williams, having thus satisfied himself, and, as he seems to hope, his pupils, that "erysipelas is a contagious and infectious disease, and consequently that it is caused by the agency of a morbid poison," calls upon them "to follow out the consequences of this admission," in the treatment of the malady. Having put the pupils in the groove, he soon shews them how they must run in it.

For, he proceeds to prove that a great many eminent doctors have entertained the most contrary notions on the best mode of treating erysipelas—many lauding bark as a specific—some denouncing all treatment save depletion as ridiculous. The modern apostle of the antiphlogistic method has been Mr. Lawrence. This gentleman does not at all mince the matter, but quietly declares that "the antiphlogistic plan, including general and local bleeding, is *the correct* view and practice; and the opinions of those who take an opposite view are completely erroneous, and the treatment founded on it not only inapplicable, but injurious." This scientific mode of settling a disputed point of practice does not appear palatable to Dr. Williams, who, not at all alarmed at Mr. Lawrence's thunder, proceeds to analyse his observations and his facts, and finds, what cannot be denied, that the former are illogical and loose, and the latter more calculated to tell against Mr. Lawrence than for him. In fact, Dr. Williams' criticisms on Mr. Lawrence's paper are very well founded, both in reasoning and practice, so far as they go; and it is a pity that he has not exposed more fully its serious faults.

It is not necessary to follow Dr. Williams, in his notice of the different modes of practice which different individuals have pursued in erysipelas. The medical world are pretty well acquainted with them. We shall content ourselves with stating the plan that Dr. Williams recommends. It is this.

"My own experience leads me to adopt a tonic mode of treatment, as the great rule in idiopathic erysipelas, but I have for some years abandoned the use of bark. For I observed that, when the patient was treated by bark, he did not become convalescent till his tongue had become brown, and, consequently, not until he had sunk into a typhoid state, that lowest point of depression consistent with life, and at which the poison either ceases to be generated, or else the system is rendered insensible to its further action. At this point, however, but not before, the bark appeared to me to take up the disease, and the patient, in a very large majority of cases, recovered."

Anxious to avoid "the brown-tongue stage," he instituted a number of experiments, and was led to the conviction that the treatment by wine was, and is superior to that by bark, or to any modification of that treatment.

"The mode, then, in which I am in the habit of treating idiopathic erysipelas, whatever may be the part of the body it attacks, or *with whatever symptoms it may be accompanied,* is as follows:—The patient is put on milk diet; the bowels gently opened, and from four to six ounces of port wine, together with sago, allowed daily. This mode of treatment it is seldom necessary to vary throughout the whole course of the disease, and the delirium, if present, is generally tranquillized, and if absent, prevented. The tongue rarely becomes brown, or only continues so for a few hours, and the local disease seldom passes into suppuration or gangrene. In a word, all the symptoms are mitigated, and the course of the disease shortened. I have pursued this treatment for several years, and I hardly remember a case in which it has not been successful.

On comparing the effects of wine and bark with those of quinine, the former is much more easy of digestion; its effects are more transient; it has less tendency to affect the head, or to disorder the bowels, and it likewise in no case reduces the patient so low, and consequently there does not exist the same necessity for supporting him. The quantity of wine may appear small, but as it is prescribed on the very first appearance of the disease, it is usually sufficient. Still, in a very few instances, it may be necessary to increase it. This was the case with a young woman admitted only a few weeks ago into this hospital, with excessive erysipelas of the face and scalp; six ounces only of wine were prescribed for this patient, and with this dose the disease showed a tendency to spread down the neck and shoulders; but on the wine being increased to eight ounces the disease was at once arrested, desquamation ensued, and the patient rapidly recovered. In another case also it would appear that a larger quantity of stimulus would have been useful. A patient in Lazarus's Ward was seized with erysipelas of the face and scalp; taking it altogether, the countenance and head generally were more remarkably swollen and enlarged, and hideously deformed, than I recollect to have ever seen. Five grains of the sulphate of quinine were ordered for this patient every four hours, together with eight ounces of wine daily. The patient under this treatment recovered. About two years after, however, he was brought to the hospital with a similar attack of erysipelas, but much milder, as was supposed, in degree. Under these circumstances, quinine alone was prescribed on this occasion, but, to our surprise, he rapidly sunk and died.

It has been imagined that when an extremity, as the leg, was hard, swollen and painful, that the mode of treatment by wine was inapplicable, and that the only resource was in incisions. The following case, however, will show a different result. An exceedingly large and corpulent lady, after a short attack of rigors, was seized with erysipelas of the leg, extending from the foot to the groin, and the whole limb was hard, tense, and incompressible; her tongue was white; she was slightly delirious in the night, and her pulse was 120. Six ounces of wine were ordered daily, and the disease was by this means checked and the pulse reduced to 110; but at the end of four days it was still formidable: two grains of quinine were prescribed, in addition to the wine, every six hours. On the following morning the fever had abated, the pulse had sunk to 90, and in a few days this patient recovered without suppuration, or other local mischief taking place in the leg.

I hope I have been able to satisfy you that the laws of the simple phlegmasiæ are totally distinct from those of inflammation, depending on the agency of a morbid poison, and that there is sufficient evidence to induce us to include erysipelas among the latter class. If so, I think you will see, from the success that has attended the treatment of the cases that have been related, that there is a singular agreement between theory and tonic mode of practice in erysipelas; and the agreement between theory and practice is the surest test and only solid basis of scientific medicine."

It must be a great comfort to a student of medicine to find his preceptors at loggerheads, on the principles and practice of physic. He is certain that let him do what he may, he has some authority to back him. In the present case, it is pleasant to observe the scientific spirit in which each party calls its opponents fools. Mr. Lawrence says complacently—" If you don't think as I do you're completely wrong"—and his adversaries decide that Mr. Lawrence's logic and practice are equally absurd.

To return to Dr. Williams. His are certainly extreme opinions.

Others have contented themselves with thinking that idiopathic erysipelas *may* be contagious. The whole tenor of Dr. Williams's arguments leads us to suppose that he deems it always so. He reasons as though it were essentially a contagious and infectious malady, and never had nor can have any other origin. If that is not his sentiment we are sure that all who read his paper will suppose so, and, indeed, if it is not, his argument loses its application and force. This is what is called in common language—"going the whole hog." There is a difficulty in getting many to suppose, that erysipelas is, under ordinary circumstances, contagious at all. We are not so sanguine as to think that many will be got to believe that it is always so. They will naturally ask how it happens that traumatic erysipelas, which cannot be imagined to arise, in the majority of cases, from contagion, runs a course so precisely like that of the idiopathic; and how it happens also that both forms usually prevail at the same time? If these questions are answered, many others will follow, perhaps more difficult to reply to still; and, if Dr. Williams is inclined to write, we foresee a series of interesting letters, as numerous, perhaps, as those which have lately passed between his namesake and Dr. Wilson Philip.

Granting, however, Dr. Williams's premiss, we do not see how his conclusion follows from it. If idiopathic erysipelas be a malady of specific origin, that certainly forms an à priori argument against the propriety of bleeding. But we do not conceive that it forms any argument in favour of the exhibition of wine. Measles is a disorder, which has its source in a special contagion, yet we do not treat measles by bark or stimulants. Scarlet fever is a complaint of a similar description, yet in the great majority of cases, scarlet fever is treated by medicines of rather an antiphlogistic character.—Even small-pox does not necessarily call for stimulants, and the great pox, we all know, has its own peculiar remedy. Analogy then, so far as it goes, if it makes against outrageous bleeding, makes equally against universal stimulation. It is in this case a two-edged bistoury which Dr. Williams pokes at Mr. Lawrence, but which Mr. Lawrence, if he feels inclined, may poke again at Dr. Williams. This fight with abstract principles, is, after all, a Laputan War; plenty of manœuvring, but very little execution.

The fact is that the treatment of each disease must be tested by experience of its own results, and not by the effects of remedies in other maladies. The facts of medicine are like those of chemistry, empirical, and analogy can no more be received as proving one than the other. Analogy might lead the chemist to suppose that potassa was a metallic oxyde; but it could not be deemed so till potassium was discovered, and after *its* discovery, analogical suppositions were unnecessary. Analogy, presuming it correct, might lead Dr. Williams to think that wine is good for erysipelas. Whether it is so, is a matter to be decided by its actual results when given for the disorder.

To those results, therefore, Dr. Williams properly appeals; for he takes, in the main, the same view of analogy that we do, and that all, accustomed to reasoning, do also. He affirms that his experience has, de facto, proved wine highly serviceable. We must distinguish between his affirmation and his proof. The latter is quite insufficient; for he only gives the particulars of three cases, and a slight allusion to two more. The position, therefore, stands on his affirmation. Dr. Williams declares that wine is *the* remedy for idiopathic erysipelas in all cases, and *with whatever symptoms it may be accompanied.* It is for the profession to decide if they approve of this uncompromising practice.

For ourselves, we are, like Beppo—

A nameless sort of person; and we feel almost ashamed to offer a temporising kind of opinion, in the midst of the vigorous practitioners about us. Yet we cannot help thinking that erysipelas is not to be treated on any one exclusive plan. We cannot help believing that we have seen cases, in which the patient was robust, and the symptoms of an inflammatory character, where antiphlogistic treatment was of use—and other cases, certainly the majority in London, where the symptoms were disposed to be low and typhoid, and where ammonia and wine were beneficial. There is, no doubt, a great difference, in different cases, in the general condition of the patient, the tint of the erysipelas, the pulse, the tongue, and so on; and it sins against our general experience of disease, if those differences are to be made of little account, and one kind of treatment, be it wine or bleeding, indiscriminately followed up. It must be remembered that, if erysipelas is not itself a phlegmasia, it occasionally gives birth, like measles, scarlet fever, and small-pox, to distinct phlegmasiæ. We have seen patients, labouring under erysipelas, die from sudden inflammation of the pleura, the pericardium, and the peritoneum. At one time, we saw a good deal of the use of bark, and then these complications were frequent.

So far as we have seen of erysipelas, and we have watched it rather closely, we are certainly disposed to rank it with the exanthemata. It is ushered in with preliminary fever, and it usually runs a pretty constant course. These circumstances shew that it is not likely to be safely cut short by powerful depletion; and so far we agree with Dr. Williams. But, on the other hand, there is a good deal of inflammatory action mixed up with it, and, as in the exanthemata, there is a natural tendency to cure, if the natural operations are not materially disturbed. The practice applicable to the exanthemata in general, appears to us to be applicable to erysipelas—to watch the case with assiduity and caution—if the patient evinces a disposition to prostration, to exhibit stimulants—if decided inflammatory symptoms supervene, to deplete. What it may be in the country we know not, but we are sure that, in town, it is more often necessary to stimulate than to lower. About the third or fourth day, the patient usually begins to flag; and then it is that ammonia, or perhaps wine, must be substituted for the salines and the mild aperients previouly exhibited. If, however, we must adopt, which fortunately we need not, an extreme practice, we would much rather cleave to Dr. Williams than to Mr. Lawrence—to wine than the lancet.

### VII. Diffused Tuberculation.

*Case.* "Sarah Creed, æt. 32, admitted into Dorcas's Ward.

June 6th, 1833.—Has always enjoyed good health. Seven years ago received a blow upon the left breast, and in about three months detected a small tumour in the part, which was occasionally very painful; for three years it did not make any material progress. It amounted to the size of a walnut, the integuments remaining healthy. In about six months from that time, ulceration took place, and the wound remained stationary for nearly a year: during this interval, she used various stimulating applications. She then came under the care of Mr. Green in this Hospital, for a period of thirteen weeks; the wound healed and she felt quite restored. Shortly afterwards a chain of tubercles, superficial, colourless, hard and painful, were observed extending from the nipple of the left breast to the boundary of the axilla; these continued to form, and ulceration of the cicatrix soon followed; the tubercles seemed to bind the skin to the subjacent muscle. She was now readmitted into the hospital under the care of Mr. Travers, at which time the whole of the left breast was absorbed, and the integuments in that situation were one mass of tubercle, irregular, livid, hard, and painful, with some minute spots of ulceration, and accompanied by a sero-purulent discharge; health but little affected. Ordered *Ferri carbon.* ʒj. *ter die. Opii,* gr. j. *o. n.* Simple applications."

Hydriodate of potass—arsenic—the liquor potassæ—the strong nitric acid, were successively used and abandoned. On the 6th of November, we find the following report.

"Disease progressing. The whole of the right breast absorbed, and the integuments, from the clavicles to the umbilicus, are now one continuous mass of tubercle, hard and contracted; the cellular membrane is quite absorbed, and the skin firmly attached to the muscles, through the medium of the tubercles; several on the neck and back are yet in an incipient state, no discoloration of the skin, but hard, small, semi-transparent, acuminated pustules appear, and when these reach the size of a small pea, they become fixed and painful, and the skin turns livid. The creosote was now tried and continued until it disagreed with the stomach."

In January, 1835, she was ordered to take the iodine in substance, half a grain of the iodine pill being given thrice daily.

" *May.*—Disease extending in every direction.

*Aug.*—The disease is now quiescent, tubercles having extended as far down as the groins ; there is a single patch of ulceration about the centre of the chest ; the swollen arm, which is hard and erythematous, has been for some time placed in a swing-box, suspended form the head of the bed.

*Dec.*—The treatment seems now beginning to influence the disease, and the part certainly looks much better. The menses suppressed for the last three months. ℞. *Acet. Morph.* gr. ss. *omni nocte. Cont. Pil. Iodin.*

*Jan.* 23, 1836.—The part now presents a very peculiar aspect from the clavicle to the umbilicus ; the structure of the integuments is entirely changed ; the surface has the appearance of cicatrices of a livid colour, uneven, hard, immoveable, the cellular membrane beneath being entirely absorbed; this new structure or diseased skin is firmly attached to the parietes of the chest and abdomen, and it is covered for the most part with scales ; there is a small ulcer at the lower margin of the sternum which looks clean but dry, and not disposed to heal. The lower part of the abdomen is scattered over with many single, and many clusters of tubercles, which are now increasing (these were formerly very painful on coming out over the chest) ; the skin is but slightly discoloured and elevated, and without pain. The back, from the cervical to the lumbar region, also the sides, are covered by flat tubercles running into each other, constituting a reddened, glazed, and elevated integument; the whole of this (where the integuments have been described as firmly fixed) callous, and free from painful sensation. The lower abdomen is very prominent, great general hardness and fullness may be felt, and there is some pain on pressure in the left iliac region. Her health and spirits are very tolerable ; she is troubled with occasional headaches, but the only complaint she makes is of pain in the chest, of a shooting pricking character, and the least excitement brings on palpitations of the heart somewhat violent, which continue for hours ; digestion good, bowels regular, pulse and tongue natural ; the catamenia restored ; the arm remains much in the same condition, and she still occasionally suffers from erythema and general irritation, but in a far less degree than formerly : she is still taking the iodine in the same small doses.

*Mar.* 3.—Better, both in health and locally. She has had a copious catamenial discharge, attended with considerable constitutional excitement. The ulceration upon the sternum is healed, but there is still some creeping ulceration in various parts of the chest. The arm is well, and of its natural size. No fresh tubercles can any where be discovered.

*Mar.* 25.—Ordered to omit the iodine."

This is an instructive case. Mr. Travers makes many valuable observations on malignant tumors in general, and on the conduct which the surgeon should pursue in doubtful cases. We are sorry that we have not space for them. But we will conclude the present Report with his remarks on the preceding case.

" The disease in Creed's case is new to my experience. The skin is implicated from the commencement with the subjacent texture, for neither can the tubercle be raised, nor the skin raised nor moved upon it ; both are fixed. The tubercle is in no degree corn or wart-like, being at first smooth and semi-transparent, like horn, at its apex, for it is conical, and some so acuminated as, at first sight, to resemble vesicles ; they afterwards become broad and flattened and coalesce in clusters, and the most central ulcerate. Gangrenous ulceration is the second mode of inflammatory action : there is always a slough at the seat of ulceration; the absorption of cellular membrane, by the close adhesion of the tuberculated skin to the parts beneath, being so complete, that no bed for granulation remains. The healing is, in fact, absorption only of the decayed parts, and a process of puckering and shrinking in those around, like the crumbling caries in bone. The hardness of the integument is equal to that of the firmest cartilage. First one mamma and then the other became absorbed, and now the whole back and front of the chest are so enmailed, while the integument of the abdomen and loins, plentifully besprinkled with the tubercle, both isolated and in clusters, binds down the abdominal muscles.

The disease having first opened with what was supposed to be a scirrhous tumour of the breast, and after the slow and imperfect healing of its ulcer, the tubercles appearing in and around the cicatrix, and in the line of the absorbents towards the clavicular and axillary glands, and the mamma becoming absorbed by adhesion of the whole integument, straitened by callous induration to the ribs, are circumstances which, taken together, remind us so forcibly of one variety of quickly destructive scirrhous cancer as almost to set scepticism at defiance. But to this variety to which I allude, of universal induration and immoveable attachment of the mamma to the ribs with tubercles of the integument scattered here and there around it, with which all are familiar, the present case bears no real resemblance, owing principally to the uniform and strongly-marked character and seat of the primary tubercle, the obliteration of cellular substance, so extensive as to convey the idea of the chest being literally skin-bound, and the intermixture of the several stages of the action, the decay by partial ulcerations, and the repair by a process of puckering of the agglomerated tubercles in livid bridles and ridges intersecting each other in every direction, and leaving hollow interspaces of a paler colour. Neither does it resemble in any degree the tuberculated sarcoma nor the insulated medullary or fungoid tubercle of the integument, nor the condylomata, sometimes seen in groups and patches of considerable extent, in various regions of the body. In its destroying and healing process, it most resembled lupus, but in this only was the analogy. The health has been, on the whole, less disturbed than could have been expected; the excessive œdema of the left arm from destruction of the superficial absorbents has exposed it to painful attacks of erythema, but this was greatly relieved by suspension of the limb. Local applications only of the simplest kind to the sore, as bread and water poultice, or the watery solution of opium and the white wash, have given any relief; the gnawing pain, attended by pungent heat and bright scarlet redness, has been at times very severe for days together; the signs of arrest of the diseased action and recovery of the part have been the general subsiding and shrinking of the tubercular elevations into a more uniform surface, in some parts glazed and in others scaly, the cessation of pain, and the darker, *i. e.* more purple hue and diminished heat of the whole. There has been frequent sickness and loss of appetite, sleeplessness, with more or less febrile irritation, but no cough, no internal pains, no considerable wasting, no symptom of established hectic, or visceral disease. Iodine was the only medicine in the use of which there was temptation to persevere; and during its exhibition for a period of many months, the change, from increasing local devastation and gradual constitutional failure, to her present state of nearly confirmed recovery, has taken place. The catamenia also have been restored. Certainly the change worked by this extraordinary medicine came to the relief of my fears, that the disease was malignant; it may yet become so, for it is a subversion of structure to a formidable extent of surface, and such changes are always in this sense dangerous."

---

### Queen's County Infirmary.*

### Report for the Year 1835. By Dr. Jacob.

Dr. Jacob enters on many circumstances connected with the extent, funds, and so forth, of the establishment, to which, as they possess only a local interest, it is unnecessary for us to advert. We shall content ourselves with extracting here and there from the report, such matters as appear to be possessed of any general interest.

*Rapid Recovery of Lunatics from Injuries.*

"Amaniac was admitted—under the influence of a superstitious delusion he effected castration within a few weeks previous to presenting himself at the Infirmary, where he arrived in a state of absolute starvation, having wandered from the County Wicklow with the hope of inducing me to amputate his hand, which he had himself endeavoured to blow off with a pistol shot, at the loss however of only one finger. He continued obstinately to refuse food for many days, though on admission he appeared in a state of almost total inanition. By the persevering administration of nutritious diet, by means of the stomach-pump, his strength was re-established; the wound went on to heal kindly; he was induced to take his food voluntarily, and was delivered to his friends in safety. I have had several opportunities of observing the extraordinary celerity with which lunatics recover from injuries calculated to endanger the lives of persons enjoying perfect health; their wounds generally unite by first intention, fractures become quickly consolidated, and they rapidly recover from the most serious casualties under very disadvantageous circumstances."

The circumstance dwelt on by Dr. Jacob, has frequently attracted observation. Probably the occupation of the lunatic's mind with a subject foreign to the corporeal injury, and his usual want of solicitude for its consequences, may account, in some measure, for his recovering so surprisingly as he sometimes does. It is certain, however, that persons who cut their throats seldom do well, although the injury may not be very formidable in itself. They frequently fall into a typhoid state, and die with erysipelas, or cellular inflammation, or secondary abscesses.

*Anomalous Hysterical Affection.*

Dr. Jacob admitted two females, whose cases the following passage will describe. The girls were young, and the affection was obviously one of those irregular hysterical or nervous maladies, which are almost as various as the combinations of the kaleidoscope.

"At frequent and irregular intervals during the day, and without any obvious cause, sudden paroxysms took place; every inspiration was effected by a spasmodic effort, and attended with a sound resembling that produced by hiccup, but infinitely louder and more shrill, so much so as to be readily heard throughout the entire house; considerable uneasiness and sense of oppression being at the same time felt at the epigastrium. The paroxysm frequently continued for an hour, and left the patient much exhausted. The pulse was accelerated, the tongue loaded, the appetite impaired, bowels confined, catamenia suppressed, and considerable tenderness experienced on pressure over the region of the stomach and liver. The administration of fetid aperients, cupping and blistering of the epigastrium, with a light nutritious diet, invariably afforded complete relief; but the disease frequently recurred in some time after the patient's discharge."

Both functional and organic affections of the stomach would seem to prevail among the lower class of Irish. Their insufficient food, and privations of all sorts, afford a satisfactory explanation of the circumstance.

Erysipelas would seem to have been common. Dr. Jacob would appear to attach much faith to the local application of the nitrate of silver.

* Dublin Journal, May, 1836.

### *Mode of treating fractured Cervix Femoris.*

"In fractures of the cervix femoris I act upon the presumption that ossific union may take place, and, as I conceive, with beneficial consequences, I have invariably found that the patients on whom most pains had been bestowed to retain the fractured portions of bone in accurate juxta-position and immoveable, ultimately recovered the most perfect use of the limb. I have succeeded to a considerable extent towards effecting this object in the following manner:—The patient being placed in bed, a strong cotton stocking, to either side of which a stout bandage had been sewed along its entire length, is drawn on the leg above the knee, it is then firmly secured on by pinching it up, and sewing it tightly when drawn as wide as it can be stretched. This being done, a very considerable degree of extension can be exercised by means of the attached bandages, the pressure of which, instead of bearing upon one point, is equally diffused over the knee, calf, ankle, and instep; the closeness with which the limb is embraced, prevents the occurrence of any degree of subsequent swelling, and the necessary degree of counter-extension can be kept up without inconvenience or danger of sloughing; the extremity is next extended to its proper length, and placed in position; the thigh is surrounded with the tin splints and cushions already described, the front one being connected with, and forming a limb of a large splint, on which chiefly depends security from motion. It consists of a shield or plate sufficiently large to cover the greater part of the anterior surface of the abdomen, the sides being hollowed for the reception of the spines of the ilia; from this a limb runs down over Poupart's ligament along the front of the thigh, which it assists to envelop; a stuffed cushion preserves all parts from pressure; the lower portion is secured with the other splints to the thigh by straps; the upper by bandages round the pelvis. A very considerable amount of counter-extension is then made by means of Desault's splint, to which the tails of the stocking bandages are tied; a thick pad is next placed high up between the thighs, and both limbs bandaged firmly together. In this way the leg and thigh of the injured side are used as a lever, the pad between the thighs serving as a fulcrum, and any pressure calculated to cause a shortening of the neck of the bone, or the entrance of the neck into the cancellated structure of the shaft prevented. The bandages running from the stocking to the Desault's splint must be kept from time to time carefully tightened. To derive real benefit from the treatment, the patient should remain from two to three months in bed, and forbear to use the limb for a much longer period. By this method I have had cases recover with little perceptible lameness, but in no instance did I succeed in preventing a slight degree of halt. A laced stocking of Indian-rubber web, with suitable bandages, may be an improvement upon what I have been in the habit of using."

Fractures of the skull are extremely frequent, a melancholy proof, as Dr. Jacob remarks, of the sanguinary spirit of the people.

### *Swallowing of Pins.*

The following observations on the effects of swallowing pins, or, indeed, other foreign substances, are, though simple, not unworthy of attention.—Patients constantly maintain that the foreign body is still sticking in the throat, at a time when the sensation is merely the effect of the irritation that some part of the œsophagus has suffered. It is frequently necessary to pass an œsophagus-bougie, and to declare that this has pushed the substance down into the stomach. But to return to Dr. Jacob.

"Amongst the list of cases is one who swallowed a pin: she came to me from an adjoining county, complaining of very distressing symptoms, and I was therefore obliged to admit her for a few days. Females to whom this accident may have occurred constantly come to me for the purpose of having the pin removed. If it so happen that it may have passed down the œsophagus, it is almost impossible to convince the patient that it does not remain in the throat. She points to the situation of either corner of the os hyoides as the seat of all her troubles ; there she feels the soreness, increased by every attempt to swallow, and there she obstinately insists she can distinctly feel the pin when she passes her finger down the throat. It may be readily supposed that her efforts soon induce a degree of inflammation, which renders the illusion more confirmed, as conjunctival vascularity induces a sensation of sand in the eye, or inflammation of the fauces a feeling of a foreign body there. On passing down the finger we may ourselves for a moment remain in doubt. The muscles of the pharynx being thrown into a state of spasmodic action, force the cornu of the os hyoides forcibly against the mucous membrane, and the sensation communicated to the finger resembles strongly what might be expected from the presence of a large pin lying across under this membrane. That this mistake, into which even a practitioner may fall, might lead to serious consequences, is proved by a case which came under my care not long since. A young girl having allowed a pin to slip down her throat, lost no time in proceeding to a neighbouring surgeon. She felt quite satisfied that she could place his finger where it lay at the butt of the tongue, as is commonly described. He examined the throat, and felt so convinced of its presence, that he made a deep incision at the side of the os hyoides for its removal; I need scarcely add, without effect. He was favoured by fortune, and escaped injuring any of the large vessels, but considerable inflammation supervened, and the girl was obliged to seek admission at the Infirmary, where she slowly recovered. Much better had he committed the harmless deception, sometimes practised by my father, of slipping into the basin a pin answering to the description of the one swallowed by the patient."

*Remarks on the Mode of using the Catheter in some Cases of Retention of Urine.*

The following hints are not undeserving of consideration. Dr. Jacob is speaking of a case of retention of urine, with numerous false passages, and a very diseased condition of the urethra. After all but himself had failed in introducing the catheter, and after he himself had not succeeded in numerous attempts, he goes on to state :—

" I carefully introduced a silver catheter of No. 11 size, which is nearly a quarter of an inch in diameter, kept it closely directed along the upper surface of the urethra, and passed it on till it hitched against the opening of one of the false passages immediately at the bulb ; I then passed my finger into the rectum, brought it firmly against the catheter, the handle of which I quickly depressed, using the instrument as a lever, my finger as a fulcrum : the point passed up behind the arch of the pubis, and entered the bladder. This manœuvre I had before tried in vain with instruments of the ordinary size. The poor man has had several returns of his complaint each year, and I have always succeeded in relieving him in this manner, latterly without difficulty, as I suppose in consequence of the false passages having become contracted, and the urethra more open by the repeated introduction of the instrument. I have by the use of the instrument, in several instances, succeeded in affording relief which other hands had failed to effect, and I have found it particularly useful in cases of retention from enlargement of the prostate gland. Within the last few months, I was called to a neighbouring county to visit a gentleman advanced in life, who had been labouring under retention for four or five days, and on whom three well-qualified practitioners had made several efforts on different days to introduce the catheter, and consequently abandoned the attempt as hopeless, and made up their minds to puncture. By the use of the large instrument, in the manner already described, I effected the desired object without either delay or difficulty. The prostate was much enlarged and indurated; a large sized gum-elastic catheter was kept in for some time, but the bladder has not yet acquired the power of emptying itself, and thus the patient is obliged frequently to introduce the instrument himself. I find that I can use a metallic instrument with more confidence than the gum-elastic, and I believe it to be safer than the latter with its stylet. I have, however, sometimes observed that the flexible instrument might be introduced when the silver one could not; it has occasionally happened that by passing on the gum-elastic instrument with a strong stylet, till it meets with the obstruction, then firmly fixing the stylet with the left hand, and urging the tube forward with the right, the stylet being kept unmoved, it will enter the bladder, though this could not be effected in any other way. I have found this plan more useful than the partial withdrawal of the stillette for the purpose of shortening the curve of the instrument, or raising it over an obstacle in the passage."

*Malignant and Hæmorrhagic Tumors.*

The following case is additionally interesting, when taken in connexion with the case of diffused tuberculation, which we have extracted from the third number of the St. Thomas's Hospital Reports.

*Case.* A female aged 45, had from infancy a small tumor about the size of a pea over the left malar bone, it was unproductive of any inconvenience, she took it off by means of ligature; it reappeared, and she a second time removed it in a similar manner fifteen years since; it did not again return, but left a small mark, to which she paid little attention. About a year previous to admission, this part began to swell immediately after she had discontinued nursing. In six weeks a tumor was formed as large as an orange, tender to the touch and fluctuating; she punctured it with a large needle, which caused an abundant discharge of blood, but of no other fluid. Shortly after ulceration set in with considerable activity, attended with frequent hemorrhage and paroxysms of severe pain; another tumor soon after formed under the left ear, of an irregular shape, hard, and the seat of severe lancinating pains. Becoming alarmed, the patient applied to a countryman who professed to cure cancer; he put a plaster on the ulcer of the face, to remain on for four months. This application induced the most violent pain, which she endured with fortitude for the prescribed period; during this time several small tumors formed in different situations, their appearance being generally preceded by a degree of sickness, the part about to be affected becoming tender and inflamed. On removing the plaster, she found that all the soft parts beneath it had been destroyed, and the cheek-bone laid completely bare. From week to week the tumors became more numerous, and her health progressively gave way.

When admitted she presented an extraordinary appearance. She was excessively emaciated. The left malar bone, and the anterior and outer third of the orbit, lay completely denuded, and presented the appearance of bones which had been exposed for half a century in a churchyard; the soft parts had cicatrized around, and lay in firm contact with the dead bone. The globe of the eye had fallen in, and little trace of it remained. The situation of almost every gland was occupied by a carcinomatous tumor. The left parotid was much enlarged, reached forward on the cheek, as high as the zygoma, and half-way down the neck; it was of almost stony hardness, very irregular on the surface, and prevented the mouth from being opened wider than half an inch. The situation of the mammæ was occupied by similar productions resembling the healthy gland in size. Several hundred tumors of various sizes, from that of an orange to a grain of snipe shot, were studded over the trunk and extremities; those on the trunk being larger than on the limbs, and those on the lower extremities larger than on the upper: they were more numerous and closely situated near to them at a distance from the centre of the circulation, and appeared in great numbers along the course of the large absorbent vessels. The liver felt enlarged and irregular as if also affected, and several hard tumors were to be felt in different situations through the abdomen. In the larger sized alone did she suffer pain, which was of a severe lancinating character, and particularly distressing during change of weather. She laboured under a most distressing sense of weight at the præcordia, her respiration was in some degree embarrassed, she had frequent and profuse perspirations, but the bowels acted naturally, and the catamenia had continued to appear from time to time at irregular periods. In a few days after her admission she was attacked with erysipelas, and left the house.

Dr. Jacob has observed two cases of what he terms hæmorrhagic tumor.

Both presented at first the appearance of cancerous warts; one was situated in the lower lip of a young female, the other connected with the external jugular vein of a man aged about forty years; their structure was highly vascular, and the tops covered with a kind of horny crust, on the removal of which blood commenced to drop quickly, and usually continued to flow for a considerable time. From that over the jugular vein it occasionally burst forth in a small jet, and continued sufficiently long to induce considerable debility. Styptic or astringent applications proved ineffectual, the knife was alone found sufficient for their cure.

---

### Glasgow Royal Infirmary.

On Phlebitis, particularly as connected with Secondary Abscesses. By James Douglas, A.M. Late House-Surgeon to the Glasgow Royal Infirmary, &c. 8vo. pp. 48. Glasgow, 1835.

This Essay was submitted to the faculty of physicians and surgeons of Glasgow, when the author was a candidate for admission into their body; but if it had no further claims on our attention than this, we should not have considered it worthy of notice.

There are few branches of medical knowledge, which have sprung up so exclusively from the recent cultivation of pathology, as our acquaintance with the phenomena and consequences of phlebitis, and with the secondary inflammations that occur after accidents or operations. The gradual progress of information on these points may be said to have occurred under our immediate inspection, as almost all that has been done for its advance has been within the last twelve years.

When it was ascertained that deposits of pus may occur in any viscus, or, in many and various parts of the body, after local injury or an operation, it was generally supposed that some strange state of the nervous system was the cause of their occurrence. But, as dissections were obtained in greater numbers, and practised with more care, and as they shewed that inflammation of the veins of the injured part very frequently preceded the secondary affections, it was concluded that phlebitis and absorption of pus were the agents that produced them.

Some plausible objections have been urged against this, as an exclusive or universal theory; and we think that the opinions of the best informed members of the profession on the subject are at present far from settled. Whilst the faith in the sole agency of the nervous system has been much shaken, the belief in the constant antecedence of phlebitis to the secondary inflammations has not yet been firmly established.

Mr. Douglas has paid much attention to this interesting and important investigation, and he advances some views, which he has been led to entertain, with a great degree of confidence. He has been anticipated in these views by Cruveilhier, but they are entitled, notwithstanding, to the careful consideration of the profession. We shall therefore lay them before our readers, so fully as to make the latter sufficiently acquainted with them.

The object of Mr. Douglas is to point out the connexion between phlebitis and the secondary inflammations, and the immediate mode in which the latter are occasioned. He describes in order: — the primary phlebitis — the secondary affection—and finally the relationship of both.

1. *Phlebitis.*

We do not think it necessary to enter on the circumstantial description of the history, the symptoms, or the anatomical characters of phlebitis. Our readers must be sufficiently acquainted with them. We shall merely select a point or two for observation.

1. After stating that phlebitis is a common consequence of injuries either to the vein itself, or to the parts in which it is situated, Mr. Douglas remarks:—" Idiopathic phlebitis has been rarely observed in the extremities, but I believe cases have occurred in which the varicose saphena has inflamed without any surgical interference, and a spontaneous cure has been the result."

It has occurred to us to witness several instances of idiopathic inflammation of the saphena vein. In one instance it followed exposure to cold—in the others it occurred without any ostensible cause. The patients were, with the exception of the one in whom cold was the apparent cause, of a bad habit of body, and in indifferent health. They all recovered, with obliteration of the canal of the saphena vein, which was felt as a hardened cord. The symptoms, which were severe, were checked by the application of leeches and blisters, and the exhibition of calomel and opium internally.

We may observe, that we once saw fatal inflammation of the saphena and the femoral vein, from what seemed to be a very slight injury. A man, rather advanced in life, and of a bad constitution, was admitted into St. George's Hospital, on account of an extensively varicose condition of the saphenæ veins. In order to relieve him, he was kept in bed, and the house-surgeon one morning tapped the vein with his fingers rather smartly. Phlebitis rapidly succeeded, and proved fatal in very few hours. A good example of the danger of even the most trivial interference with the veins in some individuals.

2. Mr. Douglas remarks that inflammation of the visceral veins is more frequent than is usually supposed, and that it wears the symptoms of simple inflammation of the organ. Andral gives two cases of inflammation of the veins of the liver. Our author has seen three, and subjoins one.

*Case.* A labourer, aged 26, admitted Nov. 19, 1833. Situated in right hypochondrium is a firm, hard tumor, about the size of an orange. It is freely moveable under integuments, and sometimes is found higher or lower in abdomen, according to the position in which patient has been for some time previously. There is some feeling of connexion to the liver, but not distinct. There is pain on pressure. Pulse 110; tongue red at the edges, but furred in the centre; bowels torpid.

Tumor was first observed eight weeks ago, after an inflammatory attack, supposed to be hepatic, at which time it was as large as now; it then increased, and finally, for the last month, has been decreasing under the use of iodine, both internally and by inunction. Slight catarrhal symptoms have supervened on his journey yesterday.

Leeches—castor-oil—mild aperients—blue pill—and cupping to the region of the liver were prescribed.

On the 26th, there being diarrhœa, the pills were discontinued, and mercurial inunction substituted for them. The diarrhœa, however, persisted—frequent leeching and cupping were necessary—and on Dec. 5, he was so low as to require whiskey and water.

On the 15th, we find him labouring under profuse sweats, and occasional rigors in the night. After this the weakness increased—the pulse remained above 100—the tongue was florid and smooth. On the 20th, there was an attack of pain at the epigastrium. In the night of the 21st, he vomited a pound of blood, and next morning had an evacuation of grumous blood. The tumor was now found to have disappeared. On the 11th of February he died.

*Dissection.* A quantity of albuminous milky fluid was found in peritoneal cavity. Liver not enlarged, its substance less yellow than natural. Internal membrane of vena portæ in all its branches inflamed and covered in most places with a layer of coagulable lymph. Some of the branches were filled with pus, and looked like small abscesses when a transverse section was made. Vena portæ healthy at its entrance into the liver, the diseased appearances stopping abruptly. The venæ cavæ hepaticæ were healthy. Gall-bladder pale, and nearly empty, the fluid in it thin, and of a pale yellow colour.

The tumor was found to consist of a mass of cancerous glands united together, placed between the layers of the mesentery, immediately below the superior transverse portion of the duodenum. Within it was a cavity more than two inches in diameter, with ragged walls, and on its left side was a communication with the duodenum immediately below the pylorus by a perforation an inch wide. Beside this, there were one or two ulcers on the pylorus, but there did not appear to be any carcinoma of the stomach.

"It is worthy of remark," says Mr. Douglas, "that the case presented the appearances of hepatitis, and was treated as such both before and after admission. Yet no mark of inflammation of the substance of the liver was found on inspection, and the pain must have resulted from the affection of the veins. The lymphatic and purulent effusions, and their abrupt commencement, exhibit the same characters as in external inflamed veins. The length to which the disease was protracted, was remarkable as compared with the generally rapid progress of traumatic phlebitis."

It is by no means so obvious to us, that the inflammation of the vena portæ can be regarded as the cause of all the pain and inflammatory symptoms. There was a malignant tumor in the mesentery, involving, of course, the visceral nerves, producing ulceration of the duodenum, and giving rise also to ulcers on the pylorus itself. Are these to be regarded as trifling affections, and to be set aside in the consideration of the causes of the symptoms? When we reflect how little local pain usually attends phlebitis even in the extremities, how frequently indeed the femoral and iliac veins are found completely choked with lymph and pus when they were not even suspected of being diseased at all, we must pause before we can agree with Mr. Douglas in the present instance. It is most probable that the phlebitis, in this case, was secondary, both in time and in importance. The acuter symptoms were no doubt induced by the ulceration of the duodenum and pylorus, and the inflammation of the vena portæ contributed only to accelerate the patient's death. Such is a fairer estimate of the circumstances than that put forth by Mr. Douglas.

3. Mr. Douglas alludes to an assertion of M. Cruveilhier's, that he has seen more fatal cases of phlebitis after trepanning operations for the removal of necrosed or otherwise diseased bone, than after any other surgical operation; and he thinks that the danger is much enhanced by repeating the unfinished operation, while the parts are in a state of inflammation.

A case occurred about a year ago in London, where fatal inflammation of the dental and maxillary veins followed the extraction of a tooth. Pus was found in the veins of the brain.

This does not at all accord with what we have ourselves seen. It has happened to us to witness a great number of cases of operations for the removal of sequestra in necrosis. At St. George's Hospital, the operation has been performed, perhaps, more frequently than at any other. Yet we do not recollect a single instance in which that operation produced phlebitis, or the secondary inflammations. This is remarkable, because there is scarcely an operation, the most trivial, that we have not seen give rise to one or the other. Of course there is no reason why this should be exempt from consequences of such common occurrence, and our not having seen it in any instance followed by them must be regarded as, in some measure, accidental. But on the other hand, we conceive that accident must have occasioned the extreme frequency of the circumstance, within the observation of M. Cruveilhier; and if we have seen it less frequently than natural, he has seen it more so.

2. *Secondary Inflammations.*

In considering these, we shall do as we have done in the examination of Mr. Douglas' remarks upon phlebitis — merely select such points as appear to us to be novel, or to require examination.

1. The following is our author's description of the anatomical characters of the secondary inflammations. His whole theory hinges on what he here states.

"Their anatomical characters present three distinct stages. The first consists in a bloody infiltration, firmer than the surrounding healthy structure, pretty accurately circumscribed, and generally assuming a globular form, except where modified by approaching the surface. In its substance, one or more minute veins full of pus may sometimes be distinguished. In the second stage, a section exhibits the inflamed portion firmer, and of a blackish colour, while in the middle there is a yellowish white purulent deposit of a circular form. The pus is not liquid, or in a free cavity, but is concrete and as if infiltrated, while the remains of the displaced tissue, like blackish cellular substance, circumscribe it into lobules. In the third stage, softening has taken place from the centre to the circumference, the cellular shreds have disappeared, and a perfect abscess has been the result. These collections, when situated at the surface of the viscera, act as irritants to the serous membranes which cover them, and a layer of coagulable lymph is generally effused over them. Two cases have been recorded, in which the inflammation ran so high that gangrene of the lung took place. I do not set this down as a fourth stage, because it does not form part of the regular course of the disease, and because it may take the place of either the second or third stage.

If attention be paid to the situation of these abscesses, they will always be found around a venous trunk, whose branches may be traced into them. When small, particularly, they may be compared to a bunch of grapes, to which the small veins serve as pedicles. These small veins, filled with pus, and with discoloured and thickened coats, may be traced into each deposit; while in the second stage and in the third, they may be traced to its edge, where they are found to end abruptly. Sometimes a vessel of considerable size is seen thus truncated.

The liver and lungs are the commonest seats of purulent deposits; next to them the spleen, kidneys, and brain. Other parts, however, are subject to them as well as the viscera, such as the muscles and the cellular tissue. In these parts, likewise, capillary phlebitis is always found."

The first case which induced our author to believe that the secondary inflammation was, in fact, capillary phlebitis of the part thus secondarily inflamed, is detailed. It is as unsatisfactory as a case, adduced to prove such a point, can be supposed to be. A man was attacked with pain in the region of the liver, for which depletion and salivation were ineffectually employed. At the end of five weeks, rigors and perspirations supervened. In twenty-six days more, a fluctuating swelling was perceptible externally. This was punctured and matter was obtained; but hectic was established, and the patient lived until nearly five months from the commencement of the symptoms. On dissection, the liver was found to contain numerous abscesses, from the size of a hazel-nut upwards, and one larger than the fist, connected to the ribs, some of which were carious. The branches of the vena portæ were inflamed, contained pus and false membranes, and terminated insensibly in the abscesses. The colon adhered to the diseased liver, and was inflamed and perforated at that part; and the psoas muscle was black from sanguineous engorgement. A quantity of serum was found in all the serous cavities.

Mr. Douglas has "no doubt that the phlebitis of the vena portæ was the primary disease." We have very great doubts upon the subject, and for the following reasons. In the first place, the case ran the ordinary course of inflammation and suppuration in the liver; and not at all the ordinary course of phlebitis. Mr. Douglas gets out of this difficulty, by supposing that the phlebitis was of a subacute character. In the second place, the fact of the branches of the vena portæ being filled with pus and lymph, offers no conclusive evidence of primary phlebitis, because, in malignant deposites in the liver, that vein is found to become filled at last with the cancerous matter.—The structure of the liver is so much made up of the ramifications of the vena portæ, that it would be singular if, when suppuration has extensively occurred in it, purulent matter was not sooner or later contained in them, and inflammatory action propagated along them. Finally, not to mention other reasons of inferior force, we must always lean, in doubtful circumstances, to the belief in the ordinary, rather than the extraordinary fact, and it is more likely that abscess in the structure of the liver, a comparatively common affection, should occur, than inflammation of the vena portæ, an uncommon one.

This was not an instance of secondary inflammation. Mr. Douglas relates a case in which such inflammation of the lungs took place. We will only extract the notice of the dissection, as that exhibits satisfactorily enough, the implication of the venous radicles in the deposits.

" There were old adhesions of pleuræ on each side. In substance of lungs were numerous purulent depôts, presenting the usual infiltrated curdy aspect. Into some of these the pulmonary veins could be traced, exhibiting thickening of their coats, and containing masses of coagulable lymph of a bright yellow colour, adhering to their inner coat. There were also two abscesses in right lung, containing each about ℨij. of liquid pus.

Liver very yellow, large, and soft; on its surface were numerous yellow spots, under its peritoneal and fibrous tunics, in the centre of each of which was a small globule of air. It was afterwards ascertained by injection, that these were the extremities of the portal veins: their coats were not inflamed."

Mr. Douglas quotes a case from M. Cruveilhier, in order to shew " one variety of phlebitis," that which takes place in bones after compound fractures. The case is short, and we cannot do better than extract it in our author's words.

*Case.* Pierre Niles, groom, aged 30, on the 29th July, 1830, received a gunshot wound in the knee-joint, comminuting the femur. Amputation was performed in the Hospital Beaujon. He went on well for twelve days, no fever, the wound healing to a wish. All at once violent rigors came on, lasting for some hours, followed by a profuse sweat, which remained all the rest of his illness. Wound became dry, covered with a grey crust, and the muscles retracted. No pain was complained of either in thorax or abdomen. Meanwhile strength sank; pulse became slow and irregular; and death took place on the 28th August, in a state of considerable emaciation, a month after the operation, and on the eighteenth day from the first rigor.

*Inspection.*—The spleen, liver, and lungs presented analogous alterations, which we may consider as three periods of the same lesion.

The spleen, smaller than usual, was here and there studded with dense spheroidal masses of a deep red. Liver presented on its surface large spots of a slate colour, which when cut, exhibited irregular masses, more or less deep, marbled with white spots, each of which was evidently formed by a glandular grain infiltrated with concrete pus. In the lungs were numerous complete abscesses.

A search was now made for primary phlebitis, but neither the veins of the stump nor those in other parts of the body presented any alteration. Upon making a longitudinal section of the remaining part of the femur, however, its cells were found filled with pus.

Let us look at the point to which our author has brought us. He affirms that where secondary inflammations follow local injuries, a double phlebitis *always* occurs: the veins of the injured part inflame first, are filled with pus, and convey it into the circulation; then, the veins of some other part, of the viscera, or the muscles, or the cellular tissue, inflame secondarily and give rise to suppuration around them. To do justice to our author we must give his conclusions a little more in detail.

"To explain," he says, "how phlebitis produces secondary abscesses, I consider myself authorised to state, that pus in the circulation is their invariable cause.

In all the dissections in which purulent deposits have been found, pus has been found in the veins, when it has been carefully sought. One or two cases must be excepted, in which the disease ran a long course, and the diseased veins had become closed by lymph through a great part of their trajet, where undoubtedly pus must have been secreted. Were it not necessary that actual pus should be in the circulation, why would not visceral abscesses take place after other primary affections? Were it otherwise, why should we not have secondary deposits after large common abscesses as well as after traumatic abscesses? We often see a large abscess disappear suddenly; the pus has been absorbed, and must be in the blood, yet there are no deposits. The reason is plain, it is altered in its absorption."

We cannot say that we perceive the force of this reasoning. It is an instance of the petitio principii. Mr. Douglas first tells us that pus in the circulation is the invariable cause of the secondary abscesses—then that a large common abscess will disappear suddenly, from absorption of the matter into the circulation—yet that no deposits take place, *because* the pus is altered in its absorption. How does he know that it is altered? By what mechanism or what process is the alteration effected? We have at present only our author's ipse dixit for it, and the alteration seems to have no other foundation than simply his opinion that it *ought* to take place. But to proceed.

"We can only conceive of three ways in which pus could get unchanged into the current of the circulation, *viz.* injection into the veins, imbibition by their open mouths, and suppuration of their cavities. Experiments, to be afterwards mentioned, prove the efficacy of the first. The second never takes place, for before suppuration comes on in a wound, the veins have adhered; and even were we to grant that they remained open, and that imbibition could take place through them, the necessary deduction would be, that deposits should occur in every case of suppurating wounds, which is a plain reductio ad absurdum. Suppurative phlebitis, then, is the only mode which fulfils the required indications, for here pus is actually secreted into the current of the blood.

I have now arrived at the same point as Mr. Arnott, and I think I have brought forward some additional facts and arguments in proof of that conclusion. I have now to go a step farther, and point out the precise manner in which pus in the blood gives rise to secondary abscesses."

Mr. Douglas alludes to the doctrine of the translation of pus, and to the opinion that although the secondary abscesses are not merely the effects of translation, they depend on some mysterious alteration of the blood. He repudiates these notions, and pledges himself to prove distinctly the three following points:—1st, that the visceral abscesses depend universally on inflammation of the minute veins in the substance of organs; 2d, that these inflamed veins produce a circumscribed engorgement and suppuration around themselves; and 3d, that the circulating pus acts by irritating the coats of these vessels, and exciting them to inflame.

1. "In every case of secondary abscess, above detailed, which I dissected since I met with the inflamed vena portæ, I have been able to trace inflammation of the minute veins of the part. These are generally very small, so that it is just possible to slit them up with the scissors. In Case VIII. it was impossible to lay open the cavity of the inflamed venous radicles in the spleen and kidney; but by scraping and washing away the surrounding substance, they were left firm and round, filled with pus, which could be squeezed out of their cut extremities. In pursuing such investigations, I cannot sufficiently inculcate minuteness and accuracy. In this same case there was no obvious primary phlebitis; yet so convinced was I of its necessary co-existence, when I had seen the visceral deposits, that I returned to the examination of the prostate, and it was only after slitting up eight or ten different branches that the diseased ones were detected.

With regard to the lungs, I am a little in doubt whether the seat of the inflammation is in the pulmonary arteries or veins. The dissections which I have already made lead me to place it in the veins;—Cruveilhier, however, states that he found the clots, and pus, and other marks of phlebitis, in the pulmonary arteries."

Our author is very confident, for he reproaches those who may differ from him on this subject with carelessness in their dissections, or with learning pathology by proxy, and promises that if they examine the bodies of the dead as minutely as he has done, they "will indubitably arrive at the same results."

2. The inflamed veins produce circumscribed engorgement, inflammation, and suppuration around themselves in the substance of organs. The name *deposits*, which has been so long applied, is unfortunate, in as much as it tends to maintain the notion which was formerly believed, that the same pus which had passed into the circulation, was again deposited from it, without the inflammation of the substance of the organs in which the deposit takes place. Now it has been already stated, under the head of anatomical characters, that the first stage of these abscesses is a sanguineous engorgement, occupying a circumscribed space, into which, by-and-by, pus is secreted, till a complete abscess is formed.

3. The most recondite portion of our author's theory is yet to come. It is that which affects to deal with the *mode* in which these secondary inflammations of the veins take place.

"The pus acts by irritating those vessels and exciting them to inflame, through which its globules probably pass with difficulty.

While passing through the larger veins, it has not time to make any impression upon them, but when it gets into the capillaries where it is retarded, and becomes applied to the lining membrane, which indeed is the only part of the vein found in the parenchyma of organs, it excites inflammation of the membrane, and consequently of the substance around it.

There is no doubt that pus, although considered a bland substance, is able to produce irritation in the vessel with which it comes in contact; the fact has been experimentally determined. Pus has been injected into the veins of animals, and phlebitis and purulent deposits have been the result. But as it was impossible to detect the precise position of the particles of pus which caused this inflammation, recourse was had to mercury, a substance which could not possibly become incorporated with any of the animal fluids. A few globules were injected into one of the meseraic veins of a dog, and on dissection, red spots were seen in the liver, some of which had proceeded to suppuration, and in the centre of each of which was seen a globule of mercury, in one of the terminal branches of the portal vein. All the branches of the vena portæ were found full of pus.

When the mercury was injected into the femoral vein, similar effects were produced in the lungs, around the globules. In a rabbit which survived for two or three months, tubercles were deposited around the globules and the abscesses. It is scarcely necessary to remark, that the rabbit is very subject to tuberculous formations.

Injection of mercury into the medullary canal of the femur, produced the same effects as when thrown into the femoral vein. Its entrance into the veins of the bone was undoubtedly favoured by the manner in which their mouths are kept open by their attachment to their bony canals. This experiment is an additional confirmation of the fact already stated, that deposits after compound fractures depend on inflammation of the diploic or cancellous veins.

From the consideration of the anatomical fact, that the pulmonary vessels are the capillary termination to the blood from the extremities, and that those of the liver are the same to the blood from the intestines, it might naturally be expected that after phlebitis in the extremities, deposits would be found in the lungs alone, and that only phlebitis of the meseraic veins would affect the liver. It does not, however, appear from observation, that the pus necessarily affects any one organ; it may pass through the lungs to the liver, and vice versa, and may obstruct veins in other viscera, or in the cellular substance, or muscles of another extremity. I have already noticed, in a case of prostatic phlebitis, that neither the lungs nor the liver were secondarily affected, but the spleen, kidneys, and substance of the heart, although lesion might have been expected in the lungs, as the prostatic plexus empties itself into the internal iliac veins."

Such is a view, indeed an ample one, of the contents of this Essay. We have laid the principal facts and opinions of Mr. Douglas before our readers, and it only remains for us to offer a few observations upon them.

From what we can gather in conversation, it would appear that many gentlemen regard the investigation of phlebitis and of the secondary inflammations, as rather amusing than practical. They affect to smile at those who pursue it, as persons of a theoretical turn, who are diverted from the study of matters of daily interest, to the pursuit of novel and fanciful pathological researches. There cannot be a more palpable blunder than this—one which stamps those who commit it as ignorant and routine men, who are totally unacquainted with the nature of many cases they daily have occasion to treat. In private as in hospital practice, the secondary inflammations are not an unfrequent cause of death, and he who does not know their characters, will be little likely to anticipate their coming, and will be confounded when they actually occur.

The secondary inflammations are so common, that an exact acquaintance with their causes is a matter of the utmost consequence. Such an acquaintance alone offers us a chance of preventing their occurrence, and prevention appears to be the only way in which we can hope to diminish their fatality.

We must distinguish the observations of Mr. Douglas into those which relate to matters of fact, and those which are essentially opinions. The facts on which he insists are these.

1. That phlebitis is the invariable cause of the secondary abscesses.

2. That those secondary abscesses are themselves the result of a secondary phlebitis, the inflammation being seated in the venous radicles.

We will begin with speaking to the first point.

When Mr. Douglas asserts in so positive a manner that *he* has always found phlebitis in those cases where secondary abscesses followed, he affirms a fact within his own immediate cognizance, and which we can therefore take upon his testimony. But we do not think that his opportunities or reading justify him in pronouncing that all who institute a careful examination must necessarily arrive at the same conclusion. M. Velpeau assures us that he has looked for phlebitis, in the presence of those who believe that it always exists, yet he has not succeeded in finding it. Great as Mr. Douglas's authority may be, accurate as his investigations have been, we cannot suppose that they are strong enough to upset M. Velpeau's statement. Granting even that pus may be found in some inconsiderable venous radicle, we are not quite sure that our knowledge of the state of the veins, in cases of ordinary suppuration is sufficiently precise to enable us to lean with all our weight on this slender circumstance. The veins certainly absorb. In cases of medullary sarcoma, it is extremely common to find the veins loaded with medullary matter. It is probable, therefore, that in most instances of suppuration, and particularly of diffused suppuration, a rigid examination would find pus both in veins and absorbents. We do not say that such *is* the case, but we do say that it is very likely, and that our ignorance upon the subject should induce us to receive such statements as these of Mr. Douglas with reserve. The positiveness he displays is exceedingly injurious to the interests of science.

There are many circumstances opposed to the reception of the opinion that phlebitis and the resorption of purulent matter must necessarily precede the visceral abscesses. Setting aside the testimony of those, who declare that they have not been able to discover any trace of inflammation of the veins, those visceral abscesses occur in cases where such inflammation can hardly be deemed possible, and where there has been no pus for resorption. We have seen cutaneous erysipelas give rise to deposites in the lungs. In many instances we have seen it followed by secondary inflammations of the pleura, pericardium, and peritoneum. These were instances merely of cutaneous erysipelas, where no suppuration whatever existed, and where phlebitis can scarcely have occurred. Intermittent fever is occasionally followed by secondary inflammations; and the same is the case in common typhus. In the latter, however, ulcerations of the mucous membrane of the bowels are frequent, and it may be urged that the small veins of the intestines may be inflamed. On the whole, we think there is great reason to doubt, if the visceral inflammations and abscesses are, of necessity, preceded by inflammation even of the small veins. Yet it is probable, that, in the majority of instances, they are so.

In the number of this Journal for April 1833, will be found some observations on this subject, which were elicited on an examination of the sentiments of M. Velpeau, in his work on Operative Surgery. As many of our present readers may not be in possession of that number, and as the observations which we made in it, tend, we think, to display the real state of the question, we will introduce a portion of them in the present place. We remarked then :—

"There are several opinions as to the causes of these depôts. One party supposes that they depend on the resorption of pus, with or without phlebitis—a second, that the resorption of pus is always connected with phlebitis — a third, that they are the result of simple idiopathic inflammations—a fourth, that they depend on some peculiar state of the nervous system. There may be other sects, but the four we have mentioned are the chief.

We need not argue the question of simple idiopathic inflammations. There are few who now entertain this opinion, and, consequently, it would be fighting with a shadow to combat it.

Is the state of the nervous system to be looked on as the cause? We were at first inclined to attribute great importance to the agency of the nervous system; we are now rather shaken in our belief. It must be confessed that it is difficult to reason satisfactorily on the agency of a system so complicated and so powerful, and it does appear to us extremely unphilosophical to get rid of that difficulty, by denying the influence of the nervous system altogether. It is not improbable that it does exercise an influence, a mysterious one indeed, in the production of the deposites. Thus we find that the patients most commonly affected with them, are persons who have lived and drunk freely, debauchees in whom the nervous system is extremely excitable, and who are subject to delirium tremens. When we think upon this fact, we cannot resist the suspicion that certain states of the nervous system are more favourable to the development of secondary inflammations than others. Beyond this, we know of no certain data for satisfactory conclusions.

If we are not content with the theory of nervous agency, we are necessarily driven to the humoral pathology. Is the blood then contaminated in these cases, and is pus absorbed or in any way carried into the current of the circulation? Let us look at facts.

Purulent depôts seldom if ever occur, unless there be a suppurating wound, or pus be locked up in some part of the body. The cases in which the secondary inflammations are most common are undoubtedly those of compound fractures, amputations, operations in which the knife passes deeply among parts, injuries of the head, and inflammation of the veins. Wherever, after an injury or an operation, inflammation and formation of lymph or pus occur in the intermuscular cellular tissue, a part in which it must *necessarily* be confined, we may count pretty generally on secondary inflammation occurring somewhere. This fact has struck us most forcibly, and so far as our own observation extends, we can guarantee its accuracy. If, on the other hand, suppuration occurs where it is not of necessity locked up, for instance, in the subcutaneous cellular tissue from which incisions discharge it, the secondary inflammations are beyond comparison less common. This is a broad fact that carries with it extreme weight.

It may be said that in chronic abscesses, in what is termed the psoas abscess for example, pus is locked up for a length of time, without much tendency to secondary inflammations existing. The cases do not appear to us to be parallel, nor the analogy sufficiently exact to diminish the force of the previous fact. A psoas abscess is connected with a peculiar state of constitution, has altogether a different march, occasions very different symptoms, from those remarked in cases of deep cellular inflammation after a recent injury or operation. No two things can be more unlike in appearance, and in all essential circumstances, than a patient during the first week or two after the reception of a compound fracture, and one with chronic abscess. In the former the inflammation is a spreading one—in the latter the purulent collection is more or less bounded by a wall of lymph.

We repeat then that the cases in which we must peculiarly dread the supervention of secondary inflammations, are those in which the inflammation has occurred in parts, where pus, if secreted, must be necessarily confined. We need hardly say that such cases must be most adapted for the resorption of pus. But there is another circumstance that cannot fail to have weight. The cases in which we might imagine, à priori, that pus might pass with much facility into the circulation, are those of phlebitis. Now two facts present themselves to our notice:—first, that phlebitis is very commonly attended with the purulent depôts, and secondly, that the symptoms of phlebitis are so similar to those of the depôts, that, accidental circumstances of distinction not being present, we defy any surgeon to point out the difference. It will be observed that this does not involve the question of the existence of phlebitis in all cases of depôts. That is a totally separate consideration.

Facts, then, have carried us thus far; first, that depôts are most common where, after injuries and operations, matter is confined, and particularly where it is not lodged in any one cavity, but disseminated through the intermuscular cellular membrane; secondly, that phlebitis is very frequently accompanied or followed by the depôts; and, thirdly, that the leading symptoms occasioned by phlebitis and by the depôts are almost exactly similar.

We know not whether we have put the argument in a clear manner, or whether our readers see its force as we have seen the force of the facts it represents. Those facts have made us swerve from an inclination to believe in the efficient operation of the nervous system, to a more than suspicion that the true cause exists in the passage of pus, in one way or other, into the circulating system. The evidence is not perfect we allow, but it is of a cumulative character, and more satisfactory than that on the side of nervous agency.

We have said that the constant existence of phlebitis in cases of depôts requires other evidence for its support. The facts to which we have alluded do not prove it. We have seen many cases of secondary inflammation, in which a careful inspection detected no venous inflammation. This has been the case with other observers. M. Velpeau has examined, since this theory was broached, thirteen patients in whom he could discover no phlebitis whatever. Some of these dissections were made in the presence of those who adhered to the theory in question. It has been said that the smaller veins are affected, and that it is difficult to trace their implication. But this is obviously assumption, and where facts fail we must stop. When the evidence of sense no longer serves us, one man has, cæteris paribus, as good a right to deny as another has to affirm.

With reference, therefore, to the coexistence of phlebitis and the purulent deposites, it seems to us that we may venture on the following conclusions, but that facts do not yet support us farther. First, that the symptoms of phlebitis and the purulent deposites are very similar, if not identical in kind. Secondly, that in a considerable number of cases, phlebitis and the purulent deposites are found, by examination after death, to co-exist. Thirdly, that in many cases a very careful inspection can detect no traces of phlebitis, while the purulent deposites are present.—Fourthly, that in many cases, phlebitis exists without the purulent deposites.

There is one circumstance that has always made a strong impression on our minds, and to which we cannot forbear adverting. It is this. If after an injury or an operation a patient has continued to have a very frequent pulse, that patient has almost always done ill, and has in many instances died of some secondary inflammation. A frequent pulse is, of course, merely an evidence of fever, and fever itself is usually a symptom, an assemblage of deranged functions from some local lesion or irritation. We do not mean to say that fever is never idiopathic, but we may safely say that in the cases we are treating of it seldom is so.

It appears then that if, after an injury or an operation, that description of fever which is characterized by a pulse always frequent, but more so at night, and a temperature of the surface always above par, be continued for any length of time, the patient is very liable to be attacked by some secondary inflammation. We are not solicitous about the explanation of facts, provided the facts themselves be true. Our own impression is, that if, after any local injury, fever from any cause be maintained, that very fever becomes itself the source of other affections. The blood is circulating and continues to circulate in all the organs with unusual rapidity, and it is not difficult to conceive that under such circumstances those organs should be more prone to inflammation. There are some analogies that bear upon this point. It is well known, for instance, that during the re-actions that follow any kind of collapse, visceral inflammations are not uncommon.—Thus a patient receives a severe burn, there is much collapse, stimulants are given to a great extent, considerable re-action succeeds, and fatal inflammation of the pleura or peritoneum supervenes. Mr. Earle mentions that many of the firemen burnt during the conflagration of Covent Garden Theatre, died from visceral inflammations. They were much stimulated. Now in these cases the secondary inflammations occur in some instances before suppuration has commenced in the injured parts, and consequently before absorption of pus can occur. But persons labouring under intermittent fevers are liable to serous or pneumonic inflammations. In intermittents there is no cognizable source of pus to be absorbed. A very common cause of death in patients who are said to have typhus, is inflammation of the lung. We have seen a patient die of secondary pleuritis, who had simply cutaneous erysipelas, and in whom there was no where suppuration. These facts, and we might mention many more, facts too of such a nature that any man of experience or of observation must recognize them as not uncommon, are calculated, we think, to prove that consecutive inflammations occur under conditions, and in diseases agreeing only in this, that there is fever.

We repeat that we are not solicitous about an explanation, nor do we feel anxious respecting the fate of our *opinions*. All that we covet is the power of observing facts correctly, and drawing from them the fair inductions they allow. Were we inclined to press our theory, or rather generalization, for it scarcely deserves to be termed a theory, we might instance the ulcerations of the bowels, the chronic pleurisies, the tubercles even occurring in protracted febrile states of system, whether the pyrexia be occasioned by a suppurating burn, diseased bone, diseased joint, or in many other manners. We will, however, desist, contented with hinting our belief, that consecutive diseases, whether they be visceral inflammations rapidly fatal, or merely chronic, are apt to occur whenever pyrexia is present for any length of time, whatever the cause of that pyrexia may have been.

Such were our sentiments in 1833. In 1836, we see little reason to modify them. We have not only seen, but closely watched, very many cases of secondary inflammations. We are too well convinced of the liability to them which is conferred by collections of matter—we are well aware of the frequency of primary phlebitis, and of the probability of the resorption of pus—but we cannot agree with Mr. Douglas, in supposing that all is clear, decisive, and above-board, nor jump at the positive conclusion that he does. To doubt in such cases is the province of reason—to investigate is the consequence of doubt—and exactness and certainty are the results of investigation. Other occupations have latterly prevented us from pursuing a series of experiments and inquiries which we once commenced, and possibly never may resume. We recommend others to observe these cases closely, and, in particular, we would advise Mr. Douglas to apply the same diligence he has exhibited already, to the further elucidation of this interesting department of pathology. He is young and sanguine. We would not damp the generous enthusiasm of his youth; but we would have him pause ere he adopts or expresses uncompromising opinions. As we grow older, we all grow more cautious, if not more wise, and we learn to distrust, not only the conclusions and the facts of others, but the observations and convictions of ourselves.

The second point to which we must allude, is Mr. Douglas' statement, that the secondary abscesses originate in inflammation of the venous branches of the part, which is the seat of the secondary inflammation. Much credit is due to him for the observation of this fact. We have no doubt of its general truth. Several years ago, we observed the same thing in some cases of deposites in the lungs at St. George's. We remember tracing the pulmonary veins, and finding them inflamed and loaded with pus, in company with our friend Mr. Smith, the present lecturer on anatomy in the Hunterian Theatre of Great Windmill Street. But we looked on the circumstance as accidental, and did not pursue the examination in other cases.

But, whilst we admit the probable general correctness of the observation, we doubt if it is universally true.

It must not be forgotten, that the secondary consequences of injuries and of phlebitis are not merely abscesses in certain parts or organs, but also inflammation of the serous or synovial membranes. A patient, for example, may be affected, after a compound fracture, with collections of pus in the lungs, or the liver, or the subcutaneous cellular membrane—or he may have deposites in the lungs, and sero-purulent effusion into the peritoneum or the pericardium—or he may have solely serous inflammation—or he may have serous inflammation, with purulent collections in the larger joints. There is no end to the combinations of these secondary inflammations, and the circumstances all shew that they are analogous in character, follow the same causes, occur in the same states of constitution, and produce the same results. We cannot dissever them, and the etiology of the secondary inflammations must involve, not only the visceral abscesses, but the serous inflammations and the collections of pus in the joints.

In secondary abscess of the lung or liver, it is easy enough to suppose that the veins of the part are inflamed. But it is not so easy to account, in this manner for sero-purulent effusion into the pleura or the peritoneum, independently of visceral abscess, or to explain on this hypothesis the collections in the joints. Mr. Douglas has felt this difficulty severely, and has resorted to an expedient more ingenious than satisfactory. He asserts that, in the articular affection, sympathy is the cause of the inflammation; an example so obviously tainted with theory, that it does not require elaborate discussion.

Again, then, we must pause, before we can subscribe to what is no other than a dogma on the part of Mr. Douglas—that the secondary abscesses result from consecutive phlebitis. This is sometimes the case, no doubt; but, in the instances of the joints and of the serous membranes, we possess no proof of its occurrence. And when Mr. Douglas soars to explain even the *mode* of the secondary inflammation, by declaring that the pus circulating in the blood becomes an irritant to the venous radicle—when he revives, in short, the doctrine of an error loci, he should be made aware how far he is departing from that precision of generalization and of reasoning, so necessary in all scientific investigations, and so especially required in this.

On endeavouring to sum up what Mr. Douglas has done, we think it must be owned that he has added fresh force to the sentiments of those who seek for an explanation of the secondary inflammations in the entrance of pus into the circulation. If we have disagreed with him on some points, it has been rather that we have doubted than actually denied his positions. We are sure that he has displayed both zeal and ability, and we augur well of those qualities applied so early to the investigation of a complex subject.

